# Identification of a UDP‐glucosyltransferase conferring deoxynivalenol resistance in *Aegilops tauschii* and wheat

**DOI:** 10.1111/pbi.13928

**Published:** 2022-10-13

**Authors:** Rizky Pasthika Kirana, Kumar Gaurav, Sanu Arora, Gerlinde Wiesenberger, Maria Doppler, Sebastian Michel, Simone Zimmerl, Magdalena Matic, Chinedu E. Eze, Mukesh Kumar, Ajla Topuz, Marc Lemmens, Rainer Schuhmacher, Gerhard Adam, Brande B. H. Wulff, Hermann Buerstmayr, Barbara Steiner

**Affiliations:** ^1^ Department of Agrobiotechnology (IFA‐Tulln), Institute of Biotechnology in Plant Production University of Natural Resources and Life Sciences, Vienna Tulln Austria; ^2^ Laboratory of Plant Breeding Department of Agronomy, Faculty of Agriculture, Universitas Gadjah Mada Yogyakarta Indonesia; ^3^ John Innes Centre Norwich Research Park Norwich UK; ^4^ Department of Applied Genetics and Cell Biology, Institute of Microbial Genetics University of Natural Resources and Life Sciences, Vienna Tulln Austria; ^5^ Department of Agrobiotechnology (IFA‐Tulln), Institute of Bioanalytics and Agro‐Metabolomics University of Natural Resources and Life Sciences, Vienna Tulln Austria; ^6^ Core Facility Bioactive Molecules: Screening and Analysis University of Natural Resources and Life Sciences, Vienna Tulln Austria; ^7^ Faculty of Agrobiotechnical Sciences Osijek Josip Juraj Strossmayer University of Osijek Osijek Croatia; ^8^ Department of Agronomy Michael Okpara University of Agriculture Umudike Umudike Nigeria; ^9^ Department of Genetics & Plant Breeding CCS Haryana Agricultural University Hisar (Haryana) India; ^10^ Center for Desert Agriculture, Biological and Environmental Science and Engineering Division (BESE) King Abdullah University of Science and Technology (KAUST) Thuwal Saudi Arabia

**Keywords:** *Aegilops tauschii*, deoxynivalenol, UDP‐glucosyltransferase, Fusarium head blight, *Triticum aestivum*, wheat

## Abstract

*Aegilops tauschii* is the diploid progenitor of the wheat D subgenome and a valuable resource for wheat breeding, yet, genetic analysis of resistance against Fusarium head blight (FHB) and the major *Fusarium* mycotoxin deoxynivalenol (DON) is lacking. We treated a panel of 147 *Ae. tauschii* accessions with either *Fusarium graminearum* spores or DON solution and recorded the associated disease spread or toxin‐induced bleaching. A *k*‐mer‐based association mapping pipeline dissected the genetic basis of resistance and identified candidate genes. After DON infiltration nine accessions revealed severe bleaching symptoms concomitant with lower conversion rates of DON into the non‐toxic DON‐3‐O‐glucoside. We identified the gene *AET5Gv20385300* on chromosome 5D encoding a uridine diphosphate (UDP)‐glucosyltransferase (UGT) as the causal variant and the mutant allele resulting in a truncated protein was only found in the nine susceptible accessions. This UGT is also polymorphic in hexaploid wheat and when expressed in *Saccharomyces cerevisiae* only the full‐length gene conferred resistance against DON. Analysing the D subgenome helped to elucidate the genetic control of FHB resistance and identified a UGT involved in DON detoxification in *Ae. tauschii* and hexaploid wheat. This resistance mechanism is highly conserved since the UGT is orthologous to the barley UGT *HvUGT13248* indicating descent from a common ancestor of wheat and barley.

## Introduction

Fusarium head blight (FHB) is one of the most destructive diseases of wheat (*Triticum aestivum* L.), leading to severe losses in both yield and crop quality. Most importantly, causative fungi of the genus *Fusarium* are notorious producers of mycotoxins that accumulate in the grain, constituting a serious threat to food and feed safety (Pestka, 2010). Among them, the type‐B trichothecene deoxynivalenol (DON) is the most prevalent (Bottalico and Perrone, 2002; Goswami and Kistler, 2004). The EU and many other countries have enacted maximum tolerated levels of DON in grain‐based food commodities (Knutsen *et al*., 2017). Hence, controlling this disease is of pivotal importance and growing resistant varieties is a sustainable, environment‐friendly and cost‐effective approach.

Resistance breeding and research are complicated by the quantitative nature of the resistance influenced by environmental factors (Buerstmayr *et al*., 2020). Numerous resistance quantitative trait loci (QTL) were mapped and are distributed over all 21 wheat chromosomes. Yet, only a handful of QTL has been validated across studies and successfully deployed in breeding programs (Buerstmayr *et al*., 2020; Liu *et al*., 2019; Löffler *et al*., 2009) and very few studies reported the cloning of a causal gene (Gadaleta *et al*., 2019; Li *et al*., 2019; Rawat *et al*., 2016; Su *et al*., 2019; Wang *et al*., 2020). Isolation projects for the highly effective and best‐studied FHB resistance QTL, *Fhb1*, delivered contradicting results, and the underlying molecular mechanism is still unknown (Lagudah and Krattinger, 2019; Li *et al*., 2019; Rawat *et al*., 2016; Su *et al*., 2019). In general, isolation and functional validation of resistance genes in wheat are challenging due to its vast genome size and polyploidy.

Recently, strategies have been developed to rapidly map traits in the diploid goatgrass *Aegilops tauschii* Coss., which is the donor of the D subgenome of hexaploid wheat (genomes AABBDD) (McFadden and Sears, 1946). Based on 242 sequenced *Ae. tauschii* accessions, Gaurav *et al*. (2022) established a *k*‐mer‐based association mapping pipeline, identified candidate genes for disease and pest resistance and demonstrated their transfer to wheat by transgenesis and wide crosses. *Aegilops tauschii* constitutes a valuable genetic resource for gene discovery and wheat breeding, including the genetic improvement of disease resistance (Anikster *et al*., 2005; Börner *et al*., 2015; Kishii, 2019; Zhou *et al*., 2021), since 75% of the diversity found within the D subgenome was lost in the initial hybridization events that gave rise to wheat landraces and is therefore not part of the *T. aestivum* genome (Gaurav *et al*., 2022).

Evaluation of FHB resistance in *Ae. tauschii* accessions revealed a broad variation, including a few resistant accessions that originated mainly from regions bordering the Caspian Sea that are receiving high levels of annual rainfall (Brisco *et al*., 2017). Several studies investigated the potential of the D subgenome donor, *Ae. tauschii*, for improving FHB resistance in wheat via synthetic hexaploids (Mujeeb‐Kazi, 2001a, 2001b; Oliver *et al*., 2005; Szabo‐Hever *et al*., 2018). The FHB severities varied depending on the *Ae. tauschii* and tetraploid genotypes involved and comprised highly resistant synthetics. Szabo‐Hever *et al*. (2018) compared the synthetic hexaploid wheat lines with their tetraploid parents and found an average disease reduction of 18.3% in the synthetics, suggesting that the D subgenome may play a role in reducing disease severity in hexaploid lines.

Resistance against the major *Fusarium* mycotoxin DON is an important FHB resistance component, since DON is phytotoxic and acts as a virulence factor promoting fungal spread in wheat spikes (Jansen *et al*., 2005). Of the several mechanisms of DON detoxification that have been described (Foroud *et al*., 2019; Gunupuru *et al*., 2017), the conjugation into the non‐toxic DON‐3‐O‐glucoside (D3G) mediated by members of the family 1 of uridine diphosphate (UDP)‐glucosyltransferases (UGTs) appears to be most relevant in wheat (Kluger *et al*., 2015), but this has yet to be investigated in *Ae. tauschii*.

In this study, we exploited the *k*‐mer‐based association mapping pipeline developed by Gaurav *et al*. (2022) to dissect the genetic basis of FHB and DON resistance in *Ae. tauschii* and study the mechanism of DON detoxification into D3G in order to identify candidate resistance genes in the D subgenome donor of *T. aestivum*.

## Results

### Phenotypic variation for FHB and DON resistance of the *Aegilops tauschii* accessions

**Figure 1:**
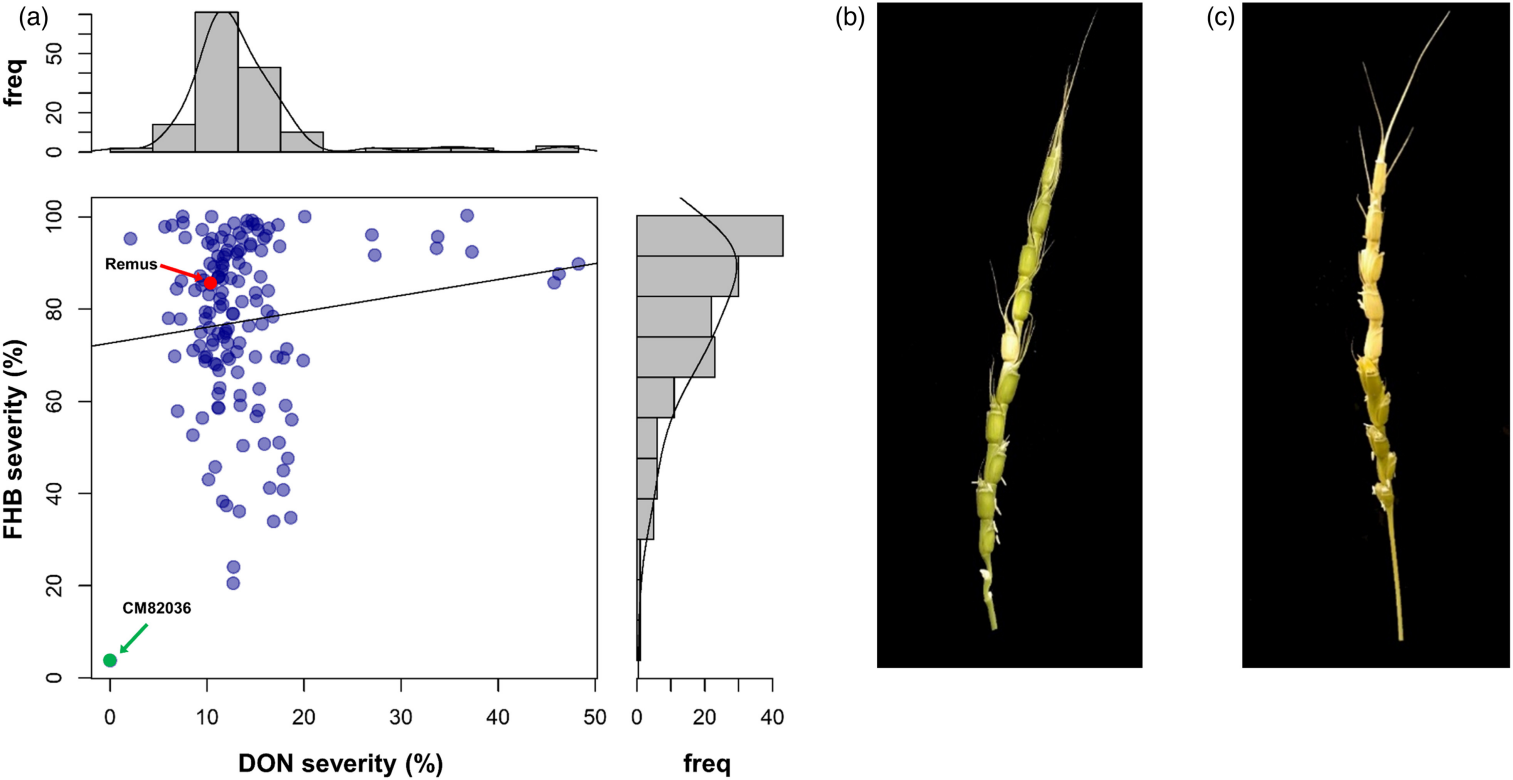
Scatter plot and marginal frequency (freq) distributions for Fusarium head blight (FHB) and deoxynivalenol (DON) severities of the 147 *Aegilops tauschii* accessions and the two hexaploid control lines CM82036 (resistant) and Remus (susceptible) (a). Severities are given in percent (%) symptomatic spikelets of best linear unbiased estimates (BLUEs) across experiments. Typical phenotypes after DON infiltration of *Aegilops tauschii* accessions with (b) low and (c) high DON severities. Note that in the resistant accession (b) only the treated spikelet showed a bleached, straw like, appearance but other spikelets stayed green, while in the susceptible accession (c) the bleaching phenotype spread.

The accessions revealed continuous variation for FHB‐ and DON‐induced bleaching severities (FHB/DON severities) (Figure 1a, Table 1), though broader variation was observed after fungal infection. FHB severities were skewed towards susceptibility with a mean of about 75% infected spikelets. The most resistant accessions developed disease symptoms on 20% of the spikelets corresponding to 1.6 symptomatic spikelets for an average spike with eight spikelets. None of the accessions was as resistant as the highly resistant hexaploid control CM82036, which did not show any disease spreading (Table 1). Regarding resistance against DON‐induced bleaching (DON resistance), most of the accessions developed the typical straw‐like colour only on the infiltrated spikelet (Figure 1b) and again none of the accessions reached the resistance level of CM82036, which did not display any DON‐induced bleaching symptoms on the spikes. Interestingly, no significant correlation was detected between FHB and DON severities (*r* = 0.1^ns^) (Figure S1).

**Table 1:** Overall means, ranges, variance components, broad‐sense heritability estimates (*H* ^2^) and least significant differences (LSD *P* < 0.05) from CM82036 (resistant control), Remus (susceptible control) and the 147 *Aegilops tauschii* accessions for: Fusarium head blight (FHB) and deoxynivalenol (DON) severities given in %, incidence for early seed shattering in the FHB and DON trials, date of anthesis, number of spikelets per spike and plant height

	FHB severity (%)	DON severity (%)	Seed shattering (FHB)	Seed shattering (DON)	Date of anthesis^†^	No. of spikelets	Plant height (cm)
CM82036	3.38	0	0	0	48.19	16.42	NA
Remus	82.78	21.45	0	0	54.07	18.14	NA
*Aegilops tauschii* accessions							
Min	20.21	0	0	0	32.50	4.40	55
Mean	76.92	14.72	30.07	1.13	46.81	8.06	99.4
Max	100	64.79	100	42.86	73.17	12.6	130
Variance ± SE							
$\sigma_{G}^{2}$	253.03 ± 40.68	44.25 ± 7.67	106.1 ± 43.71	72.69 ± 7.2e‐06	57.10 ± 7.42	3.24 ± 0.40	107.6 ± 46.9
$\sigma_{\mathrm{GY}}^{2}$	37.85 ± 25.22	0 ± 6.30	189.5 ± 52.91	5.74 ± 2.5e‐01	0.63 ± 2.10	0.23 ± 0.06	232.4 ± 56.7
$\sigma_{\mathrm{GYS}}^{2}$	109.24 ± 21.58	36.33 ± 6.34			15.15 ± 1.96	0.21 ± 0.04	
$\sigma_{\varepsilon }^{2}$	459 ± 11.99	48.47 ± 2.03	269.9 ± 33.45	0 ± 2.5e‐07	10.34 ± 0.22	0.84 ± 0.02	259.7 ± 32.5
${Η}^{2}$	0.85	0.89	0.40	0.96	0.93	0.95	0.79
LSD 5%	24.95	10.45	1.90	1.92	1.32	0.23	1.99

NA, not available.

^†^ Number of days after December 1^st^ to anthesis.

For nine accessions, the DON infiltration resulted in severe bleaching symptoms, which was mainly associated with a spreading in the acropetal direction (Table 1, Figure 1c), and even produced higher DON severities than detected for the susceptible hexaploid control Remus. The highly DON susceptible accessions originated from Azerbaijan (TOWWC0044), Turkey (TOWWC0083), the former USSR (TOWWC0088), Iran (TOWWC0097, TOWWC0134, TOWWC0137, TOWWC0168, TOWWC0169) and Armenia (TOWWC0185).

Analysis of variance revealed that all sources of variation gave rise to highly significant effects on the FHB and DON severities, although the factor genotype was most important (Table S1) resulting in high heritabilities of *H* ^*2*^ = 0.80 and *H* ^*2*^ = 0.89 for FHB and DON severities, respectively (Table 1).

The applied treatments, *Fusarium graminearum* inoculation or DON infiltration, had a strong impact on premature seed shattering and at 23 dai 36.5% of all *F. graminearum* inoculated spikes fell apart, while this was only the case for 1.6% of the treated spikes in the DON trial. In the FHB experiment, the heritability for premature seed shattering was low (*H* ^2^ = 0.4), whereas for the DON trial, the genotype was the main source of variance leading to high heritability (*H* ^2^ = 0.96). The FHB and DON severities had little influence on the seed shattering incidences and were only weakly correlated (*r* = 0.21** for FHB and *r* = 0.23** for DON) (Figure S1). Pearson's correlation coefficients of the traits for individual trials and across trials are provided in Table S2 and the BLUEs for all evaluated traits in Tables S3 and S4.

### Phenotypic variations of other traits and trait correlations

The *Ae. tauschii* accessions exhibited a large diversity for several other agronomical traits, such as date of anthesis, number of spikelets per spike and plant height (Table 1). The flowering period lasted for more than a month and the plant height varied between 55 and 130 cm, with a mean of around 100 cm. Spikelet number per spike varied between 4 to 13 spikelets. For all these traits, high heritabilities confirmed the high data quality, which was necessary for further genomic analyses. Plant height showed a weak positive correlation with FHB severity (*r* = 0.37**) and an insignificant correlation with DON severity (Figure S1). Neither flowering date nor the number of spikelets per spike was significantly correlated with FHB and DON resistance.

### The ratio of D3G to DON in toxin‐treated *Aegilops tauschii* spikes

**Figure 2:**
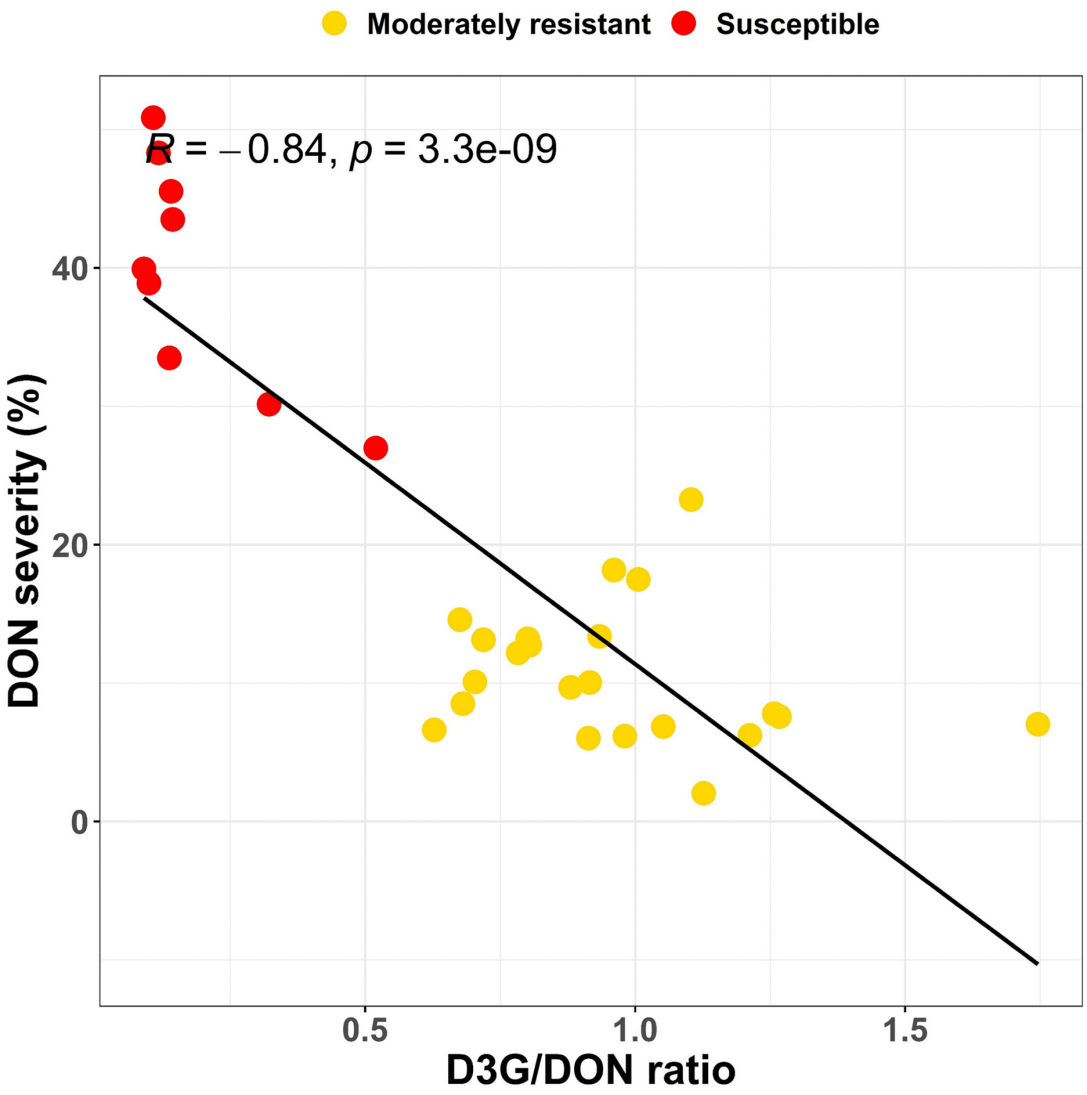
Scatter plot for 31 *Aegilops tauschii* accessions for the ratio of DON‐3‐O‐glucoside/deoxynivalenol (D3G/DON) and the DON severities evaluated in the greenhouse experiments. The accessions are coloured according to their DON severities in yellow (moderately resistant) and red (susceptible).

Thirty‐one *Ae. tauschii* accessions, including the nine most DON susceptible and 22 randomly selected moderately resistant accessions were analysed for DON and D3G contents and both were detected in all investigated accessions (Table S5). DON was found at concentrations of 0.046–1.66 * 10^3^ mg/kg (mean: 0.62 * 10^3^ mg/kg) and D3G at 0.44–2.32 * 10^3^ mg/kg (mean: 0.41 * 10^3^ mg/kg) in the DON‐treated spikes of *Ae. tauschii*. The ratio of D3G to DON varied significantly among the accessions and was strongly negatively correlated with DON severity (*r* = −0.84**), meaning that the most DON resistant accessions had the highest conversion rates of DON to D3G (Tables 2 and S5, Figure 2). In the highly resistant hexaploid control line CM82036, almost all the infiltrated DON was converted to D3G, leading to a 10 times higher amount of D3G in comparison to DON. The D3G/DON ratios of the most DON resistant *Ae. tauschii* accessions were on the other hand, close to one, while the susceptible *Ae. tauschii* accessions showed D3G/DON ratios around 0.2, which is similar to the susceptible hexaploid control line Remus (Table 2).

**Table 2:** Means and standard deviation (SD) of deoxynivalenol (DON)‐3‐O‐glucoside (D3G)/DON ratios of overall means across trials and individual trials for CM82036 (resistant control), Remus (susceptible control) and the 31 *Aegilops tauschii* accessions grouped according to their DON resistance level in moderately resistant and susceptible

Genotype	D3G/DON ratio (Mean ± SD)		
	Across trials	2019	2020
CM82036	11.0 ± 4.2^a^	15.7 ± 0^a^	8.6 ± 1.4^a^
Remus	0.32 ± 0.08^c^	0.41 ± 0^c^	0.27 ± 0.03^c^
*Ae. tauschii* (moderately resistant)	1.06 ± 0.42^b^	0.86 ± 0.21^b^	1.18 ± 0.47^b^
*Ae. tauschii* (susceptible)	0.20 ± 0.29^c^	0.14 ± 0.04^c^	0.22 ± 0.33^c^

D3G/DON ratio classes with different index letters are significantly different at *P <* 0.05 based on Fisher's least significant differences multiple comparison test.

### Association genetics for FHB and DON resistance in *Aegilops tauschii*

**Figure 3:**
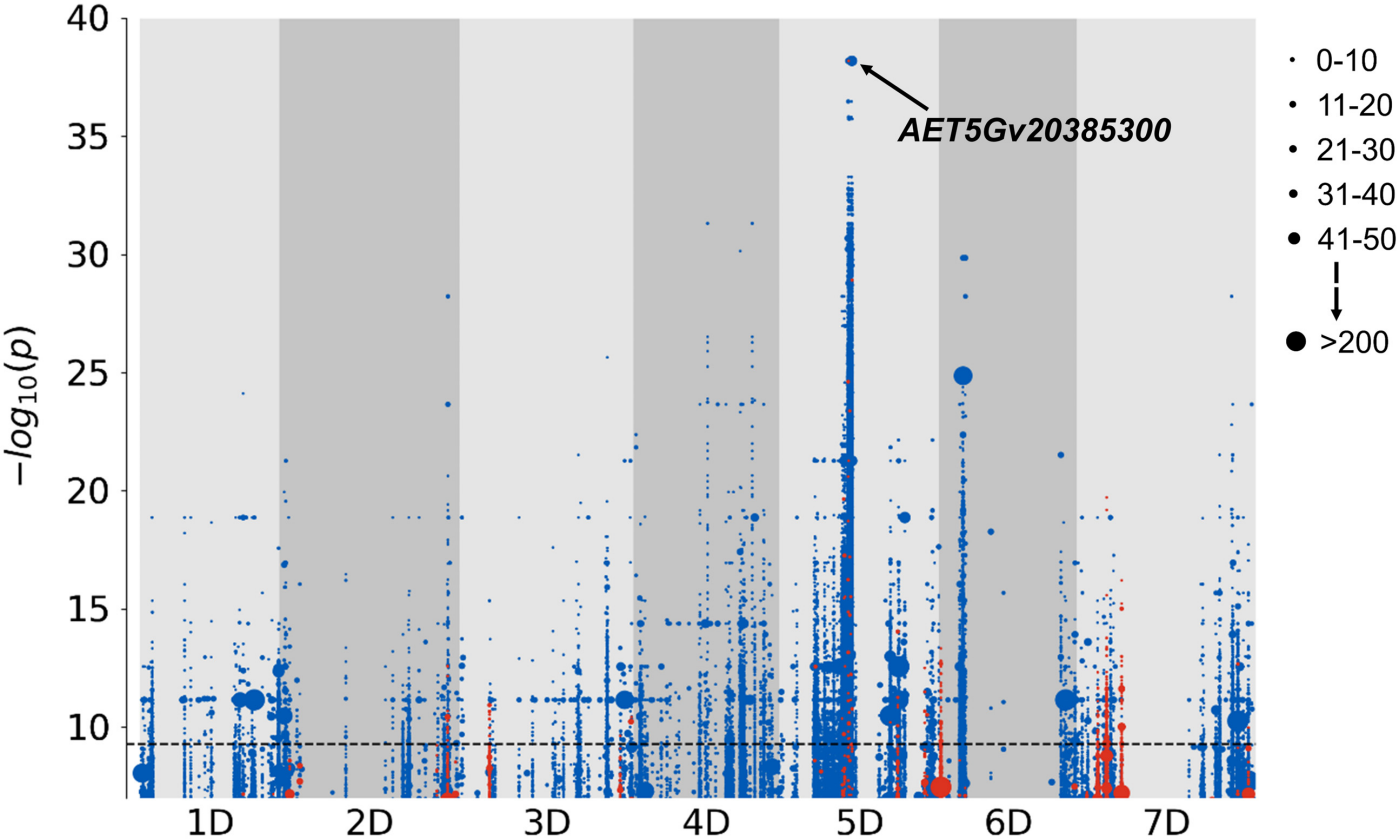
*k*‐mer‐based association genetics for deoxynivalenol (DON) severity. Points on the plot illustrate *k*‐mers associated with DON resistance (blue) or DON susceptibility (red) mapped to the *Aegilops tauschii* reference genome AL8/78 partitioned to chromosomes 1D to 7D. On chromosome 5D, the *k*‐mer comprising the uridine diphosphate (UDP)‐glucosyltransferase (UGT) *AET5Gv20385300* is highlighted. −log_10_ *P*‐value = 9.3 was taken as the Bonferroni‐adjusted threshold for evaluating the statistical significance of an association. Point size is proportional to the number of *k*‐mers with a specific −log_10_ *P*‐value (see insert).

To dissect the variation for FHB and DON resistance present in the 147 *Ae. tauschii* accessions, we used the *k*‐mer‐based association mapping pipeline based on whole genome shotgun sequence data and the *Ae. tauschii* accession AL8/78 as a mapping reference (Gaurav *et al*., 2022). For FHB resistance, many significant, minor‐effect associations were identified by analysing the across‐trials data. Most prominent was a very broad peak in the centromeric region of chromosome 7D (Figure S2), which mapped at 265.960–397.501 Mb and covered the entire pericentromeric region (Luo *et al*., 2017). This 130 Mb region contained more than 3000 genes and was significantly associated in both individual experiments (Table S6). For DON resistance a highly significant association was detected close to the centromere on the long arm of chromosome 5D mapping at 247.290 to 262.700 Mb (Figure 3). *k‐*mers (−log_10_ *P*‐value = 38.21) with strong correlations to DON severity (*r* = 0.85) mapped to two gene classes well‐known as key players in DON resistance: a UDP‐glucosyltransferase (*AET5Gv20385300*, LOC109746039, at 250.105 Mb) and two ABC transporters (ABC transporter G family member 41‐like isoform X1, *AET5Gv20401700*: LOC109787618, at 261.730 Mb; *AET5Gv20401700*: LOC109787645 at 262.022 Mb) (Table S7).

### Allelic variations of the candidate genes for DON resistance

**Figure 4:**
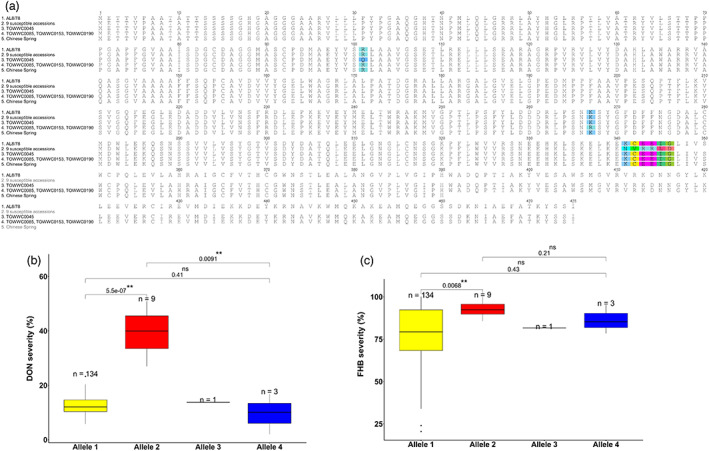
Allelic diversity at the uridine diphosphate (UDP)‐glucosyltransferase (UGT) *AET5Gv20385300* and the corresponding Fusarium head blight (FHB) and deoxynivalenol (DON) severities. (a) Amino acid sequence alignment of *AET5Gv20385300* for the four allelic variants identified among the 147 *Aegilops tauschii* accessions and the allele of wheat cv. Chinese Spring: (1) the reference AL8/78, (2) a frame shift from amino acid 341 onwards resulting in a 346 amino acid protein identified for the nine DON susceptible accessions, amino acid changes at positions 101 (3) in TOWWC0045 and at (4) 267 in three accessions (TOWWC0085, TOWWC0153, TOWWC0190). (5) Depicts the 390 amino acid sequence of the UGT allele from wheat cv. Chinese Spring. Boxplots for DON severity (b) and FHB severity (c) of the four allelic UGT variants. Significant codes: **, *P* < 0.01; ns, not significant.

Sequence alignments of the 147 phenotyped *Ae. tauschii* accessions to the AL8/78 reference identified seven mutations in the coding region of the UGT *AET5Gv20385300* resulting in four allelic protein variants (Figure 4a, Table S8): (1) the predominant allele was the 475 amino acid protein of AL8/78 found for 134 accessions; (2) nine accessions revealed a single nucleotide deletion causing a frameshift from amino acid 341 onwards, leading to a premature stop codon and consequently to a truncated 346 amino acid protein; two SNPs resulted in two nonsynonymous mutations (3) R101Q and (4) K267R detected in one and three accessions, respectively. Notably, the truncated protein was only found in the nine most DON susceptible accessions with the lowest DON to D3G conversion rates. The DON and FHB severities of the 147 *Ae. tauschii* accessions for the four UGT alleles are visualized in Figure 4b,c showing the highly significant negative effect of the deletion on DON resistance and also for FHB resistance when comparing to the majority of the accessions carrying the AL8/78 allele.

The UGT diversity analysis of the sequenced, but not phenotyped *Ae. tauschii* accessions (Gaurav *et al*., 2022) and the four *Ae. tauschii* reference sequences (Zhou *et al*., 2021) identified 21 additional mutations resulting in 11 further protein variants (Figure S3, Table S8). The most common allelic variation led to two nonsynonymous amino‐acid changes (P74A; R138Q) and was detected for 125 lineage 1 accessions; whereas all eight lineage 3 accessions possessed three nonsynonymous changes (A278V; V409L; R410C). None of the accessions showed the truncated 346 amino acid protein associated with DON susceptibility, yet one accession (TOWWC0291) carried a point‐mutation (S211*) causing a truncated, 210 amino acid protein.

Interestingly, the hexaploid wheat cv. Chinese Spring UGT allele (*TraesCS5D01G143300*) differed from all sequenced *Ae. tauschii* accessions; it carried a point‐mutation (W391*) that resulted in a premature stop codon and a truncated 390 amino acid protein compared with the 475 amino acid protein of AL8/78 (Figures 4a, S3). Four out of the 16 hexaploid wheat genome assemblies (Appels *et al*., 2018; Walkowiak *et al*., 2020) (Claire, Norin61, SY Matthis, Robigus) also held this point‐mutation. The 11 other hexaploid wheat genotypes revealed the AL8/78 allele (ArinaLrFor, Cadenza, CDC Landmark, CDC Stanley, Jagger, Julius, LongReach Lancer, Mace, Paragon, PI190962 (spelt wheat), Weebill 1) as did the two control lines CM82036 and Remus (Table S8). The homoeologous genes on chromosomes 5A (*TraesCS5A01G149600*) and 5B (*TraesCS5B01G148300*) showed high sequence similarities to the ‘5D UGTs’ (*TraesCS5D01G143300; AET5Gv20385300*) (97.4%; 96.2% and 95.9%; 94.7% identity, respectively), possessed the full‐length 475 amino acid alleles at both loci (Figure S4) and were highly conserved among the 16 hexaploid wheat lines. Only spelt wheat (PI190962) carried a nonsynonymous mutation (E192A) at the 5A locus. The durum wheat reference Svevo possessed full‐length alleles at the 5A (*TRITD5Av1G113020.3*) and 5B (*TRITD5Bv1G093730.2*) homoeologous UGTs, which were identical to the major‐allele found in wheat.

Sanger sequencing confirmed the UGT alleles of the 31 accessions analysed for DON and D3G, Chinese Spring and the two control lines CM82036 and Remus.

For the two ABC transporters, only intron variants and one synonymous mutation (T113T) for *AET5Gv20401700*: LOC109787645 were found to be polymorphic between the resistant and the susceptible classes. Based on the lack of functional variation, these genes most likely do not play a role in DON resistance at this locus and therefore, we focused on the UGT candidate gene for functional analysis.

### *AET5Gv20385300* expression confers resistance against DON in *S. cerevisiae*

**Figure 5:**
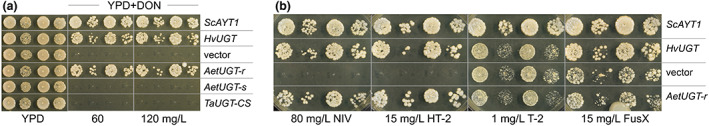
Functional evaluation of the uridine diphosphate (UDP)‐glucosyltransferase (UGT) *AET5Gv20385300* identified in *Aegilops tauschii*. A toxin‐sensitive yeast strain was transformed with plasmids expressing the AL8/78 allele of *AET5Gv20385300* (*AetUGT‐r*) and the two truncated allelic variants identified in *Aegilops tauschii* (*AetUGT‐s*) and in Chinese Spring (*TaUGT‐CS*) as well as the yeast acetyltransferase *ScAYT1* and the barley UGT *HvUGT13248* (*HvUGT*) as positive controls and the empty plasmid (vector) as negative control. Two individual transformants of each clone were grown in selective medium to mid‐log phase, diluted to OD 0.3 and 0.03 and spotted onto yeast‐extract‐peptone‐dextrose (YPD) media containing the indicated concentrations of (a) deoxynivalenol (DON) and of (b) nivalenol (NIV), HT‐2 toxin (HT‐2), T‐2 toxin (T‐2) and fusarenone X (FusX).

We used heterologous expression of the different alleles of the candidate DON detoxification gene in a toxin sensitive baker's yeast strain for functional testing. The full‐length AL8/78 *AET5Gv20385300* allele was custom synthesized and codon‐ optimized for yeast and from this template, the other alleles were constructed by *in vitro* mutagenesis (sequences in Figure S5); all variants were cloned behind the constitutive *ADH1* promoter. The empty vector served as negative control, and the barley *HvUGT13248* and the yeast acetyltransferase *ScAYT1* were used as positive controls. Whereas the full‐length allele conferred increased DON resistance, the truncated versions were nonfunctional (Figure 5a). The *Ae. tauschii* UGT behaved like its barley orthologue *HvUGT13248*, being active with both DON, nivalenol and HT‐2 toxin, but inactive against C4 acetylated toxins (T‐2 toxin, fusarenone X) (Figure 5b) (Michlmayr *et al*., 2018).

The highly similar homoeologous UGTs on wheat chromosomes 5A (*TraesCS5A01G149600*) and 5B (*TraesCS5B01G148300*) were also tested in yeast for their DON‐detoxifying abilities. The strain expressing the ‘5A UGT’ tolerated DON at the highest concentration tested (120 mg/L) similar to the *AET5Gv20385300* transformant and the positive controls *HvUGT13248* and *ScAYT1*. The transformant expressing the ‘5B UGT’ was more sensitive to DON and its growth was already retarded at 30 mg/L and almost fully inhibited at 120 mg/L DON (Figure S6).

## Discussion

FHB resistance can be dissected into several components (Mesterházy *et al*., 1999) of which resistance to initial infection (also called type 1 resistance) and resistance to fungal spreading (type 2) (Schroeder and Christensen, 1963) are the most common evaluated mechanisms (Buerstmayr *et al*., 2020). Another resistance component is described as insensitivity of wheat genotypes to DON or ability to degrade DON (Lemmens *et al*., 2005; Wang and Miller, 1988). However, DON resistance can also be regarded as a component of type 2 resistance, since DON‐deficient *F. graminearum* mutants with a disrupted trichodiene synthase gene cannot spread within the spike (Bai *et al*., 2002). Lemmens *et al*. (2005) directly assessed this resistance component by infiltration of DON solution into wheat florets. They found severe DON‐induced bleaching only in susceptible lines, while resistant lines showed high conversion rates of the applied toxin into D3G and this ability to detoxify DON into D3G coincided with the major FHB resistance QTL *Fhb1*.

In this study, we evaluated 147 *Ae. tauschii* accessions for resistance against DON and *Fusarium* spreading, as these resistance components are considered less affected by environmental factors, more stable across environments and can be more easily studied in the glasshouse (Buerstmayr *et al*., 2020). In general, FHB resistance assessment in wild relatives of wheat is challenging due to unfavourable agronomic properties such as brittle rachises resulting in seed shattering, small spikes, stiff glumes and weak straw. This makes glasshouse experiments laborious, whereas testing such plant material in field trials is, at best, very difficult but oftentimes not feasible. We adapted our phenotyping protocol and injected *F. graminearum* spores or DON directly through the tough glumes to maximize the chance of infection and DON absorption. Early seed shattering complicated FHB phenotyping as more than one‐third of the fungus‐treated spikes fell apart during the assessment period and symptomatic spikelets were lost. However, the continuous evaluation of symptomatic and total spikelets per spike allowed us to record all symptomatic spikelets, including those lost due to early shattering. This procedure gave a complete, high‐quality dataset for FHB spreading and DON resistance showing high heritabilities and strong correlations across the trials for the individual traits.

### *Aegilops tauschii* germplasm holds resistance against *fusarium* spreading controlled by many small‐effect QTL

We observed a wide range of phenotypic variation for FHB spreading resistance in the *Ae. tauschii* panel, including a few resistant and many highly susceptible accessions. Brisco *et al*. (2017) also found broad variation and a higher frequency of susceptible genotypes in their evaluation of 109 *Ae. tauschii* accessions with single floret inoculation. The 21 accessions assessed in both studies showed a similar response to fungal infection (*r* = 0.7), and the two most resistant accessions, TA2477 (TOWWC0162) and TA1691 (TOWWC0127) studied by Brisco *et al*. (2017) were also among the most resistant accessions in our trial. In contrast to the previous studies (Brisco *et al*., 2017; Szabo‐Hever *et al*., 2018), our most resistant accessions did not reach the highly resistant hexaploid control. In general, FHB severities were higher in our experiments, most likely since we used a highly aggressive isolate.

FHB resistance levels were independent from date of anthesis, number of spikelets per spike and only weakly correlated with plant height in our study, confirming that type 2 resistance is less biased by external factors (Buerstmayr *et al*., 2020). Plant height was weakly positively associated with FHB severities, which is in contrast to the usually observed relationship, that taller genotypes tend to be more resistant (Buerstmayr *et al*., 2020; Klahr *et al*., 2007). Plant height is considered as a passive FHB resistance factor affecting the likelihood of fungal infections under natural disease pressure or spray inoculations. Testing for spreading resistance using single floret inoculation, an increased resistance of taller plants has accordingly not been observed (He *et al*., 2016; Srinivasachary *et al*., 2009; Yan *et al*., 2011). Two key plant height and heading date determinants located on the D subgenome, namely the *Rht‐D1* and *Ppd‐D1*/*Rht8* loci, which frequently coincide with FHB resistance QTL in wheat (Buerstmayr *et al*., 2020) were thus not detected in this study.

The contribution of the D subgenome to FHB resistance in wheat is evidenced by many mapped resistance QTL distributed over all seven chromosomes, although compared with the A and B subgenome, the QTL number is smaller, with about 20% of all detected QTL (Buerstmayr *et al*., 2020; Venske *et al*., 2019). The D subgenome is known for its lower genetic diversity (Pont *et al*., 2019; Zhou *et al*., 2020), resulting in fewer polymorphic markers and underestimation of the contribution of the D subgenome to FHB resistance in wheat.

In this study, only the potential of the D subgenome to confer FHB resistance was analysed and on a much broader genetic diversity, resulting in many significant associations. The most significant and consistent effect mapped to a 130 Mb linkage disequilibrium block in the pericentromeric region of chromosome 7D, where several FHB resistance QTL in wheat have also been identified e.g. in the resistant cultivar Catbird (Cativelli *et al*., 2013), the resistant landrace Haiyanzhong, (Li *et al*., 2011) and the susceptible cultivars Riband (Draeger *et al*., 2007) and Nanda2419 (Lin *et al*., 2006). These findings suggest the presence of (a) gene(s) controlling fungal spread in the centromeric region of chromosome 7D in *Ae. tauschii* and in wheat, but the QTL region comprised too many candidate genes for further analysis.

### DON resistance is conferred by conjugation to D3G controlled by a UGT on chromosome 5D

The *Ae. tauschii* accessions revealed low variation for DON‐induced bleaching after toxin infiltration, except for nine highly susceptible accessions that exhibited severe bleaching. Although DON acts as a virulence factor that promotes fungal spread in wheat spikes (Jansen *et al*., 2005), DON and FHB spreading resistance were not significantly correlated. Nevertheless, our results show that DON resistance is a prerequisite for fungal spreading resistance as no accessions that were both FHB resistant and DON susceptible were identified (Figure 1a).

Different molecular mechanisms may provide DON resistance, such as sulphation, acetylation and glutathione‐mediated detoxification, as recently demonstrated by the identification of the *Fhb7* gene (Wang *et al*., 2020). Yet, evidence from metabolomics studies (Kluger *et al*., 2015) indicated that glycosylation seems the most relevant detoxification mechanism in wheat. Conversion of DON into D3G, which has diminished ability to inhibit protein biosynthesis *in vitro* (Poppenberger *et al*., 2003), is mediated by members of the very large family 1 of UDP‐glycosyltransferases, which consists of about 150–200 members in diploid grasses (Caputi *et al*., 2012; Schweiger *et al*., 2013), not all of which are functional. Despite the expectation that there should be high redundancy in UGTs with substrate specificity for DON, individual genes seem to play essential roles. In *Brachypodium distachyon*, loss of function of a single UGT gene, *Bradi5g03300*, led to increased susceptibility to DON and reduced *Fusarium* resistance (Pasquet *et al*., 2016). Likewise, an ethyl methane sulphonate‐induced mutation in *HvUGT13248* reduced DON resistance and *Fusarium* spreading resistance in barley (Bethke *et al*., 2020).

Using the *k‐mer* association genetics, we identified the highly significant UGT *AET5Gv20385300* in the centromeric region of chromosome arm 5DL as the major factor influencing DON resistance in *Ae. tauschii* in this study. The susceptible accessions showed thereby a clearly distinct haplotype: a single nucleotide deletion resulted in a truncated protein and caused more than three times increased DON severities and five times lower D3G/DON ratios. When expressed in toxin‐sensitive yeast, the truncated version of the UGT did not confer resistance against DON, whereas the full‐length allele exhibited strong activity against DON, nivalenol and HT‐2 toxin similar to the *HvUGT13248* detected in barley (Li *et al*., 2017; Schweiger *et al*., 2010). *HvUGT13248* efficiently detoxified DON (Schweiger *et al*., 2010) and nivalenol into the non‐toxic nivalenol‐3‐O‐β‐d‐glucoside (Li *et al*., 2017), and when expressed in transgenic wheat, it conferred resistance against fungal spreading and transformants converted DON more efficiently to D3G (Li *et al*., 2015).

The *AET5Gv20385300* gene identified in this study is orthologous to the barley UGT *HvUGT13248*, which shows high sequence similarity (90% identity) and is located close to the centromere of chromosome 5H indicating a highly conserved DON resistance mechanism tracing back at least 11 million years radiated from a common ancestor of *Ae. tauschii*/wheat and barley (Huang *et al*., 2002).

### *AET5Gv20385300* detoxifies DON to D3G in *Aegilops tauschii* and hexaploid wheat

The 16 publicly available whole‐genome wheat sequences (Appels *et al*., 2018; Walkowiak *et al*., 2020) and the two control wheat lines were analysed to explore the sequence diversity of the *AET5Gv20385300* orthologs in hexaploid wheat. Two allelic variants were identified: 13 genotypes carried the resistant ‘AL8/78 allele’, but Chinese Spring and four additional genotypes (Claire, Norin61, SY Matthis, Robigus) possessed a truncated version of this UGT. This UGT allele (*TraesCS5D01G143300*) is private to hexaploid wheat, it was not detected in *Ae. tauschii* and it is not functional for DON detoxification as revealed by the yeast assay.

For these 16 wheat lines no DON resistance data are available, but regarding FHB resistance, both allelic variants comprise genotypes classified as susceptible (e.g. Jagger, Cai and Bai, 2014; Robigus, Muhovski *et al*., 2012) or moderately resistant (e.g. Chinese Spring, Ma *et al*., 2006; Cadenza, Fabre *et al*., 2019) or resistant (e.g. Arina, Draeger *et al*., 2007; Norin61, Shimizu *et al*., 2021).

FHB resistance is a truly quantitative trait and DON resistance is one component to which *AET5Gv20385300* and its orthologous UGT in wheat most likely contribute.

Moreover, the highly similar homoeologous UGTs on wheat chromosomes 5A (*TraesCS5A01G149600*, 96.2% identity) and 5B (*TraesCS5B01G148300*, 94.7% identity) have similar, redundant functions. Interestingly, despite their high sequence similarities distinct differences in their DON‐detoxifying abilities were revealed by the yeast test with the ‘5A UGT’ being as effective against DON as *AET5Gv20385300* and the ‘5B UGT’ the least efficient.

The low sequence diversity of the ‘chromosome 5 UGT group’ within the 16 wheat lines suggests that these UGTs add to the general ability of DON detoxification in wheat, all wheat genotypes, including FHB susceptible ones, can metabolize DON into D3G as summarized by Lemmens *et al*. (2016) and shown for *Ae. tauschii* in this study. The detailed characterization of these loci on a broader set of wheat lines with known FHB/DON resistance levels and DON/D3G ratios will help to clarify their roles in DON detoxification and FHB resistance. Notably, a genome‐wide transcriptome analysis of 96 European winter wheats revealed that all three homoeologous UGTs were up‐regulated after *Fusarium* inoculation and thus involved in the wheat/Fusarium interaction (Buerstmayr *et al*., 2021).

Regarding FHB/DON resistance QTL on wheat chromosome 5D: the Korean wheat cultivar Chokwang was found to carry a type 2 resistance QTL on chromosome arm 5DL (Yang *et al*., 2005) and in the Chinese landrace Wangshuibai, DON content and resistance to initial infection were affected by chromosome arm 5DL (Yu *et al*., 2008). Although the closest markers to these QTL were 200 Mb distal to *AET5Gv20385300*, the large linkage blocks generated in bi‐parental mapping studies, especially in the centromeric regions, do not allow conclusions about the contribution of *AET5Gv20385300* to these resistance loci.

Overall, this study demonstrated the applicability of *k*‐mer based association mapping to dissect the genetic control of the quantitatively inherited FHB resistance and identified a DON resistance QTL on chromosome 5D. A mutation in the UGT gene *AET5Gv20385300* at this locus affects the ability to counteract the *Fusarium* virulence factor DON by conjugation into D3G. Hence, the truncated version of this UGT (*TraesCS5D01G143300*) present in hexaploid wheat genotypes presumably increases toxin susceptibility and consequently *Fusarium* susceptibility, which can be considered in genomic breeding approaches when selecting for FHB resistance.

Focusing the analyses on the D subgenome donor *Ae. tauschii* enabled thus a strong complexity reduction in comparison to hexaploid wheat and showed a great merit to further deepen our knowledge about the complex genetic control of FHB resistance.

## Experimental procedures

### Plant material

A total of 147 *Ae. tauschii* accessions were provided by the John Innes Centre, Norwich, UK (Table S9). The accessions were a subset of the *Ae. tauschii* diversity panel studied by Arora *et al*. (2019) and Gaurav *et al*. (2022) and based on whole‐genome sequencing, 113 of the 147 accessions were classified as non‐redundant. The phylogenetic grouping assigned all accessions to the *Ae. tauschii* lineage 2 (L2). The majority of the accessions originated from collection sites around the Caspian Sea; most descended from Iran (58) and Azerbaijan (57), followed by Armenia (7), Georgia (6), Turkmenistan (4), Turkey (4), Uzbekistan (2), Russia (2), the Syrian Arab Republic (1), the former USSR (1) and five accessions were of unknown origin. Additionally, two hexaploid wheat lines, CM82036 and Remus, were included in this study as resistant and susceptible controls (Buerstmayr *et al*., 2002). CM82036 is a highly FHB and DON resistant derivative of the spring wheat cultivar Sumai3 originating from China. It carries the two major FHB resistance QTL *Fhb1* and *Qfhs.ifa‐5A*. On the contrary, Remus is a susceptible spring wheat cultivar developed at the Bavarian State Institute for Agronomy in Freising, Germany.

### Glasshouse experiments and resistance assessments

Two independent experiments were carried out in the glasshouse of the University of Natural Resources and Life Sciences, Vienna, Department of Agrobiotechnology. The first experiment was set up as an un‐replicated trial during winter 2018/2019, whereas, the second experiment in the winter of 2019/20 was laid as a randomized complete block design with two replications.

Seeds of each accession were sown in multi‐trays in a mixture of heat‐sterilized compost and sand and stratified before germination at 4 °C, 12 h day/night light regime for 1 week. Thereafter, the seeds were germinated at 22°C and at the one leaf stage vernalized for 11 weeks. Five seedlings per accession were planted in 4‐L pots (18 cm diameter, 21 cm height) filled with a mixture of heat‐sterilized compost, peat, sand and rock flour. The pots were randomly placed in the glasshouse and maintained at a temperature of 14/10 °C day/night with 12 h photoperiod for the first 40 days. At spike emergence, the temperature was increased to 22/18 °C day/night with a 16 h photoperiod at 15 000 lx. For both experiments, two pots per accession were planted. In the first experiment (2018/19), one pot was used either for FHB or DON resistance assessments. In the second experiment (2019/20), in each of the two pots, spikes of three plants were inoculated with *F. graminearum* conidia suspension and spikes of the remaining two plants were infiltrated with DON solution.

### FHB resistance assessments

A macroconidia suspension of the *F. graminearum* single spore isolate ‘IFA 65’ was prepared and stored in aliquots at −80 °C as described in Buerstmayr *et al*. (2002). Shortly before inoculations, the frozen aliquots were thawed at 37 °C and diluted to a concentration of 50 000 conidia/mL with distilled water. At anthesis, 7–10 spikes per pot were inoculated by pushing an injection needle through the stiff glumes of one central spikelet to insert 20 μL of inoculum into the cavity of the florets (wounding method). The inoculated spikes were covered for 24 h with translucent polyethylene bags sprayed with distilled water to ensure high humidity for fungal infection. The total number of spikelets and the number of *F. graminearum*‐bleached spikelets per spike were recorded at anthesis and at 7, 11, 15, 19, 23 and 27 days after inoculation (dai). During the disease assessment period premature seed shattering occurred and was monitored for each spike by subtracting the number of spikelets per spike at each evaluation time point from the total number of spikelets per spike counted at spike anthesis.

### DON resistance assessments

DON (purity >98%) was purified according to Altpeter and Posselt (1994) and Lemmens *et al*. (2005) and diluted for the infiltration to a concentration of 25 mg/mL in 0.1% Tween20. In the first experiment, five flowering spikes per pot were infiltrated by directly pipetting 10 μL DON solution between the palea and lemma of the two basal florets of one central spikelet, resulting in the application of 0.5 mg DON per spike. In the second experiment, we infiltrated 20 μL of DON solution into each of three spikes per pot using the ‘wounding method’ described above. Subsequently, spikes were covered with water‐sprayed bags for 24 h. The total number of spikelets per spike and the number of DON‐bleached spikelets per spike were counted at anthesis and at 19, 23, 27 and 31 days after infiltration (dai). As explained before, pre‐mature seed shattering was recorded. At the ripening stage, DON‐treated spikes from each accession were harvested for DON and DON‐3‐glucoside (D3G) measurements.

### Plant height

In the spring and fall 2020 the 147 *Ae. tauschii* accessions were again grown in the ‘IFA‐Tulln glasshouse’ in two replications each as described for the resistance assessment trials. Before ripening, plant height was measured for each pot resulting in four height measurements per accession.

### Deoxynivalenol and deoxynivalenol‐3‐glucoside measurements

DON and D3G contents were analysed from 31 accessions infiltrated with DON comprising the nine most DON susceptible and 22 randomly selected moderately resistant accessions. Treated spikes per replication were pooled, ground and 100 mg per sample were analysed. Samples were extracted as described earlier in Doppler *et al*. (2019), using methanol/acetonitrile/H_2_O (1.5/1.5/1) + 0.1%FA (v/v) as extraction solvent (Doppler *et al*., 2016). The samples were analysed with liquid chromatography—high‐resolution mass spectrometry (LC‐HRMS). For this, a Vanquish UHPLC system coupled to a QExactive HF Orbitrap mass spectrometer (both Thermo Fisher Scientific) was used as described in Sauerschnig *et al*. (2017) with the only change in applying a shorter chromatographic method (20 min total run time; 0–1 min: 10% B, 1–11 min: 35% B, 11–14 min: 100% B, 14–16‐5 min: 100% B; 16.5–20 min: 10% B). DON and D3G standards for LC‐HRMS analysis were acquired from Romer Labs GmbH, Tulln, Austria. Quantification was carried out via external calibration using solutions of pure DON and D3G in methanol/acetonitrile/H2O (1/1/2) + 0.1%FA (v/v) at concentration levels between 1 and 5000 μg/L. Data evaluation was conducted with the vendor software Xcalibur (Thermo Fisher Scientific). In both experiments the same 31 *Ae. tauschii* accessions as well the two control lines CM82036 and Remus were analysed.

### Statistical analysis

The percentage of symptomatic spikelets at the individual evaluation time‐points, the number of spikelets per spike and the date of anthesis were analysed using a linear mixed model, which considered subsampling of individual spikes within each pot as follows:

$$Y_{\text{ijkl}}=\mu +g_{i}+e_{j}+{\mathrm{ge}}_{\mathrm{ij}}+r_{\mathrm{jk}}+p_{\mathrm{ijk}}+\varepsilon_{\text{ijkl}}$$

where $Y_{\text{ijkl}}$ denotes the observation of the individual spikes, μ is the grand mean and $g_{i}$ is the genetic effect of the ith accession. The environment effect $e_{j}$ is defined as the effect of the jth year and the genotype‐by‐environment interaction is described by ${\mathrm{ge}}_{\mathrm{ij}}$. $r_{\mathrm{jk}}$ is the effect of the 𝑘th replication within the jth year, $p_{\mathrm{ijk}}$ the effect of the ith pot within the kth replication and jth year and $\varepsilon_{\text{ijkl}}$ is the residual term.

Plant height in cm and seed shattering incidence in % were analysed with the following linear mixed model:

$$Y_{\mathrm{ijk}}=\mu +g_{i}+e_{j}+{\mathrm{ge}}_{\mathrm{ij}}+r_{\mathrm{jk}}+\varepsilon_{\mathrm{ijk}}$$

where $Y_{\mathrm{ijk}}$ is the phenotypic record of the ith accession tested in the jth year in the kth replication. μ is the grand mean and $g_{i}$ is the genetic effect of the ith accession.

The environment effect $e_{j}$ is defined as the effect of the jth year and ${\mathrm{ge}}_{\mathrm{ij}}$ describes the genotype‐by‐environment interaction. $r_{\mathrm{jk}}$ is the effect of the kth replication at the jth year and $\varepsilon_{\mathrm{ijk}}$ is the residual term.

Analysis for both models was performed with R 3.5.1 (R Core Team, 2020) using the package *sommer* 4.0.8 (Covarrubias‐Pazaran, 2016) with all effects considered as random, except $g_{i}\;$which was modelled as a fixed effect to obtain the Best Linear Unbiased Estimates (BLUEs).

To calculate BLUEs across years for FHB and DON severities expressed in %, the scoring time‐point combination with the highest correlation between the 2 years and the broadest variation for the traits was selected.

Heritabilities were estimated by assigning all effects as random and using the variance components following the formula (Holland *et al*., 2003):

$${Η}^{2}=\sigma_{G}^{2}/\left(\sigma_{G}^{2}+\sigma_{\mathrm{GY}}^{2}/y+\sigma_{\mathrm{GYS}}^{2}/\mathrm{yr}+\sigma_{\varepsilon }^{2}/\mathrm{yrs}\right)$$

where $\sigma_{G}^{2}$ is the genetic variance, $\sigma_{\mathrm{GY}}^{2}$ the genotype‐by‐year interaction, $\sigma_{\mathrm{GYS}}^{2}$ the interaction of genotype‐by‐year‐by‐replication, $\sigma_{\varepsilon }^{2}$ the residual variance component, y the number of years, r the number of replications and s the number of treated spikes.

Multiple paired *t*‐tests were performed to compare BLUEs across trials of DON and FHB severities for candidate gene alleles using the R package *ggpubr* 0.2.4 (Kassambra, 2019).

### Association genetics analysis

Whole‐genome shotgun sequences of the *Ae. tauschii* accessions were previously generated under the aegis of the Open Wild Wheat Consortium (http://www.openwildwheat.org/). Genome‐wide association mapping was carried out based on sub‐sequences (*k*‐mers) following the procedures described in Arora *et al*. (2019) and Gaurav *et al*. (2022). The reference genome assembly of *Ae. tauschii* subsp. *strangulata* accession AL8/78 (Luo *et al*., 2017) was used for genome alignments and comparative annotation.

As described in Gaurav *et al*. (2022), −log_10_ *P*‐value = 9.3 was taken as the Bonferroni‐adjusted threshold for evaluating the statistical significance of an association. Association mapping plots were generated using Python 3.7 (plotting script available at https://github.com/wheatgenetics/owwc).

### Allelic diversity analysis of the candidate genes

The 31 *Ae. tauschii* accessions with contrasting DON severities and DON and D3G LC‐HRMS measurements were evaluated for their allelic constitutions at the identified candidate genes. Scaffolds of *de novo* sequence assemblies of the accessions (Gaurav *et al*., 2022, DOIs 10.5281/zenodo.4430803, 10.5281/zenodo.4430872 and 10.5281/zenodo.4430891) were mapped to contigs of the 5D AL8/78 reference sequence comprising the candidate genes UGT (*AET5Gv20385300*: LOC109746039) and two ABC transporters G family member (*AET5Gv20401700*: LOC109787618, *AET5Gv20401700*: LOC109787645). Sequence alignments were generated with Geneious version 8.1 (https://www.geneious.com).

The diversity analysis of the UGT candidate gene was extended to the 301 sequenced *Ae. tauschii* accessions, including the set of 242 non‐redundant accessions (Gaurav *et al*., 2022) and the four additional *Ae. tauschii* reference genomes AY61, AY17, XJ02 and T093 (Zhou *et al*., 2021).

The Chinese Spring reference sequence (Appels *et al*., 2018), 10 chromosome pseudomolecules and five scaffold assemblies of hexaploid wheat (http://www.10wheatgenomes.com) (Walkowiak *et al*., 2020) were explored to analyse the sequence diversity of the UGT among hexaploid wheat and among the homoeologous UGT copies on the A and B genome. Furthermore, the Svevo durum wheat reference sequence (RefSeq Rel. 1.0) (Maccaferri *et al*., 2019) was used to evaluate the homoeologous UGT genes on the A and B genome in *T. durum*.

The UGT haplotypes of the 31 *Ae. tauschii* accessions analysed for DON and D3G, Chinese Spring and the control lines CM82036 and Remus were also generated by Sanger sequencing using the following PCR primer combination UGT5D‐F 5′ TGGAAAATGTCGGCCACTCT and UGT5D‐R 5′ TCCGGTGTGAGCTGTGAATC.

### Functional testing of *AET5Gv20385300* and the homoeologous UGTs on wheat chromosomes 5A and 5B in *Saccharomyces cerevisiae*

Synthetic versions of *AET5Gv20385300*, *TraesCS5A01G149600* and *TraesCS5B01G148300* were produced which were codon‐optimized for expression in yeast (GenScript; clone U425QGD160_2, clone U936HE240_2, clone U936HE240_4, respectively, Figures S5, S7). The synthesized DNAs U425QGD160_2, U936HE240_2 and U936HE240_4 were digested with HindIII and NotI and the inserts containing the UGTs were ligated to the HindIII‐NotI digested backbone of pWS1921/pYAK7 (Li *et al*., 2017; Poppenberger *et al*., 2003), yielding pGW1259 (*AET5Gv20385300*), pGW1300 (*TraesCS5A01G149600*) and pGW1301 (*TraesCS5B01G148300*), respectively. These plasmids contain the synthetic genes c‐terminally fused to the c‐myc epitope under the control of the strong constitutive Sc*ADH1* promoter and terminator. The truncated versions of *AET5Gv20385300* were produced by PCR using pGW1259 as template and the following primer pairs: ADHfw (CATTGTTCTCGTTCCCTTTCTTCC) + AetUGTdelAet_rv (ATTATT**GCGGCCGC**TCAGTCCAATCTTCTCACATTTTCTTTCAGTTCC) or ADHfw + AetUGTdelCS_rv (ATTATT**GCGGCCGC**TAGTGCGGAATACCAACCAGCG), respectively. The PCR fragments were digested with HindIII and NotI and cloned into the pWS1921 backbone. The resulting plasmids (pGW1260 and pGW1266) expressed the UGT proteins as present in the DON susceptible *Ae. tauschii* accessions and in Chinese Spring, respectively (Figure S5).

To test the functionality of *AET5Gv20385300*, *TraesCS5A01G149600* and *TraesCS5B01G148300* against trichothecenes all plasmids were transformed into the DON sensitive *S. cerevisiae* strain YZGA515 (Poppenberger *et al*., 2003) together with the following control plasmids: pBP910 (empty plasmid, Poppenberger *et al*., 2003), pWS1921 (*HvUGT13248*, Li *et al*., 2017) and pHW74 (*ScAYT1* (https://www.yeastgenome.org/locus/S000003986) in pWS1921 backbone). Transformants were grown in selective medium to mid‐log phase, diluted to OD600 of 0.3 and 0.03, respectively, and spotted onto yeast‐extract‐peptone‐dextrose (YPD) plates (1% yeast extract, 2% peptone, 2% dextrose, 2% agar) containing varying concentrations of DON, nivalenol, T‐2 toxin, HT‐2 toxin and fusarenone X.

## Conflicts of interest

The authors declare that they have no conflict of interests.

## Author contributions

BS, HB, BBHW, SA, KG, GW and GA planned and designed the research. RPK, BS, ML, SZ, MM, CEE, MK and AT performed the glasshouse experiments, GW and GA the testing of the UDP‐glucosyltransferase in yeast and MD and RS analysed deoxynivalenol and DON‐3‐O‐glucoside contents. RPK, KG, BS and SM performed data analysis. BS, RPK and GA wrote the draft of the manuscript. BBHW, KG, SM and SA revised the manuscript.

## Supporting information


**Figure S1** Pearson's correlation coefficients of the *Aegilops tauschii* accessions for Fusarium head blight and deoxynivalenol severities and other traits.
**Figure S2** *k*‐mer‐based association genetics for Fusarium head blight severity.
**Figure S3** Amino acid alignment of the allelic diversity at the UGT for the sequenced *Ae. tauschii* accessions and wheat cv. Chinese Spring.
**Figure S4** Amino acid alignment of the chromosome ‘5D UGTs’ and the homoeologous proteins at chromosomes 5A and 5B of Chinese Spring.
**Figure S5** Sequence of the codon‐optimized *Ae. tauschii* UGT *AET5Gv20385300*, the deduced protein sequence and primers used for *in vitro* mutagenesis.
**Figure S6** Functional evaluation of the homoeologous uridine diphosphate (UDP)‐glucosyltransferases (UGT) at chromosomes 5A (*TraesCS5A01G149600*), 5B (*TraesCS5B01G148300*) and 5D (*TraesCS5D01G143300*, *AET5Gv20385300*).
**Figure S7** Sequences of the codon‐optimized UGTs *TraesCS5A01G149600* and *TraesCS5B01G148300*.

**Table S1** Analysis of variance for the evaluated traits.
**Table S2** Pairwise correlations of the evaluated traits.
**Table S3** Best linear unbiased estimates of the evaluated traits in 2019 and 2020.
**Table S4** Best linear unbiased estimates of the evaluated traits across years.
**Table S5** Deoxynivalenol (DON) and DON‐3‐O‐glucoside (D3G) contents and the ratio of D3G/DON.
**Table S6** The mapped region significantly associated with Fusarium head blight resistance on chromosome 7D.
**Table S7** The mapped region significantly associated with deoxynivalenol resistance on chromosome 5D.
**Table S8** Allelic diversity of the uridine diphosphate (UDP)‐glucosyltransferase (UGT) for the 305 *Ae. tauschii* accessions and 18 hexaploid wheat lines.
**Table S9** List of phenotyped *Ae. tauschii* accessions.

## References

[pbi13928-bib-0002] Altpeter, F. and Posselt, U.K. (1994) Production of high quantities of 3‐acetyldeoxynivalenol and deoxynivalenol. Appl. Microbiol. Biotechnol. 41, 384–387.

[pbi13928-bib-0003] Anikster, Y. , Manisterski, J. , Long, D.L. and Leonard, K.J. (2005) Resistance to Leaf Rust, Stripe Rust, and Stem Rust in Aegilops spp. in Israel. Plant Dis. 89, 303–308. 30795354 10.1094/PD-89-0303

[pbi13928-bib-0004] Appels, R. , Eversole, K. , Feuillet, C. , Keller, B. , Rogers, J. , Stein, N. , Pozniak, C.J. *et al*. (2018) Shifting the limits in wheat research and breeding using a fully annotated reference genome. Science, 361, eaar7191. 30115783 10.1126/science.aar7191

[pbi13928-bib-0005] Arora, S. , Steuernagel, B. , Gaurav, K. , Chandramohan, S. , Long, Y. , Matny, O. , Johnson, R. *et al*. (2019) Resistance gene cloning from a wild crop relative by sequence capture and association genetics. Nat. Biotechnol. 37, 139–143. 30718880 10.1038/s41587-018-0007-9

[pbi13928-bib-0006] Bai, G.H. , Desjardins, A.E. and Plattner, R.D. (2002) Deoxynivalenol‐nonproducing *fusarium graminearum* causes initial infection, but does not cause disease spread in wheat spikes. Mycopathologia, 153, 91–98. 12000132 10.1023/a:1014419323550

[pbi13928-bib-0007] Bethke, G. , Huang, Y. , Hensel, G. , Wyant, S. , Li, X. , Morrell, P. , Kumlehn, J. *et al*. (2020) The barley UDP‐glycosyltransferase UGT13248 is required for deoxynivalenol conjugation and type 2 resistance to Fusarium head blight. In Proceedings of the 2020 National Fusarium Head Blight Forum( Canty, S. , Hoffstetter, A. and Dill‐Macky, R. , eds), p. 63. East Lansing, MI: U.S. Wheat & Barley Scab Inititiative.

[pbi13928-bib-0008] Börner, A. , Ogbonnaya, F.C. , Röder, M.S. , Rasheed, A. , Periyannan, S. and Lagudah, E.S. (2015) Aegilops Tauschii introgressions in wheat. In Alien introgression in wheat( Molnár‐Láng, M. , Ceoloni, C. and Doležel, J. , eds), pp. 245–271. Cham: Springer International Publishing.

[pbi13928-bib-0009] Bottalico, A. and Perrone, G. (2002) Toxigenic Fusarium species and mycotoxins associated with head blight in small‐grain cereals in Europe. Eur. J. Plant Pathol. 108, 611–624.

[pbi13928-bib-0010] Brisco, E.I. , Brown, L.K. and Olson, E.L. (2017) Fusarium head blight resistance in Aegilops tauschii. Genet. Resour. Crop. Evol. 64, 2049–2058.

[pbi13928-bib-0011] Buerstmayr, H. , Lemmens, M. , Hartl, L. , Doldi, L. , Steiner, B. , Stierschneider, M. and Ruckenbauer, P. (2002) Molecular mapping of QTLs for Fusarium head blight resistance in spring wheat. I. Resistance to fungal spread (Type II resistance). Theor. Appl. Genet. 104, 84–91. 12579431 10.1007/s001220200009

[pbi13928-bib-0014] Buerstmayr, M. , Steiner, B. and Buerstmayr, H. (2020) Breeding for Fusarium head blight resistance in wheat—Progress and challenges. Plant Breed. 139, 429–454.

[pbi13928-bib-0015] Buerstmayr, M. , Wagner, C. , Nosenko, T. , Omony, J. , Steiner, B. , Nussbaumer, T. , Mayer, K.F.X. *et al*. (2021) Fusarium head blight resistance in European winter wheat: insights from genome‐wide transcriptome analysis. BMC Genomics 22, 470. 34167474 10.1186/s12864-021-07800-1 PMC8228913

[pbi13928-bib-0016] Cai, J. and Bai, G. (2014) Quantitative trait loci for Fusarium head blight resistance in Huangcandou × ‘Jagger’ wheat population. Crop Sci. 54, 2520–2528.

[pbi13928-bib-0018] Caputi, L. , Malnoy, M. , Goremykin, V. , Nikiforova, S. and Martens, S. (2012) A genome‐wide phylogenetic reconstruction of family 1 UDP‐glycosyltransferases revealed the expansion of the family during the adaptation of plants to life on land. Plant J. 69, 1030–1042. 22077743 10.1111/j.1365-313X.2011.04853.x

[pbi13928-bib-0019] Cativelli, M. , Lewis, S. and Appendino, M.L. (2013) A Fusarium head blight resistance quantitative trait locus on chromosome 7d of the spring wheat cultivar catbird. Crop Sci. 53, 1464–1471.

[pbi13928-bib-0020] Covarrubias‐Pazaran, G. (2016) Genome‐assisted prediction of quantitative traits using the R Package sommer. PLoS One, 11, e0156744. 27271781 10.1371/journal.pone.0156744 PMC4894563

[pbi13928-bib-0021] Doppler, M. , Kluger, B. , Bueschl, C. , Schneider, C. , Krska, R. , Delcambre, S. , Hiller, K. *et al*. (2016) Stable isotope‐assisted evaluation of different extraction solvents for untargeted metabolomics of plants. Int. J. Mol. Sci. 17, 1017. 27367667 10.3390/ijms17071017 PMC4964393

[pbi13928-bib-0022] Doppler, M. , Kluger, B. , Bueschl, C. , Steiner, B. , Buerstmayr, H. , Lemmens, M. , Krska, R. *et al*. (2019) Stable isotope‐assisted plant metabolomics: investigation of phenylalanine‐related metabolic response in wheat upon treatment with the fusarium virulence factor deoxynivalenol. Front. Plant Sci. 10, 1137. 31736983 10.3389/fpls.2019.01137 PMC6831647

[pbi13928-bib-0023] Draeger, R. , Gosman, N. , Steed, A. , Chandler, E. , Thomsett, M. , Srinivasachary, S.J. , Buerstmayr, H. *et al*. (2007) Identification of QTLs for resistance to Fusarium head blight, DON accumulation and associated traits in the winter wheat variety Arina. Theor. Appl. Genet. 115, 617–625. 17607557 10.1007/s00122-007-0592-3

[pbi13928-bib-0024] Fabre, F. , Bormann, J. , Urbach, S. , Roche, S. , Langin, T. and Bonhomme, L. (2019) Unbalanced roles of fungal aggressiveness and host cultivars in the establishment of the fusarium head blight in bread wheat. Front. Microbiol. 10, 2857. 31921038 10.3389/fmicb.2019.02857 PMC6917580

[pbi13928-bib-0025] Foroud, N.A. , Baines, D. , Gagkaeva, T.Y. , Thakor, N. , Badea, A. , Steiner, B. , Bürstmayr, M. *et al*. (2019) Trichothecenes in cereal grains – an update. Toxins, 11, 634. 31683661 10.3390/toxins11110634 PMC6891312

[pbi13928-bib-0026] Gadaleta, A. , Colasuonno, P. , Giove, S.L. , Blanco, A. and Giancaspro, A. (2019) Map‐based cloning of QFhb.mgb‐2A identifies a WAK2 gene responsible for Fusarium Head Blight resistance in wheat. Sci. Rep. 9, 6929. 31061411 10.1038/s41598-019-43334-z PMC6502796

[pbi13928-bib-0027] Gaurav, K. , Arora, S. , Silva, P. , Sánchez‐Martín, J. , Horsnell, R. , Gao, L. , Brar, G.S. *et al*. (2022) Population genomic analysis of Aegilops tauschii identifies targets for bread wheat improvement. Nat. Biotechnol. 40, 422–431. 34725503 10.1038/s41587-021-01058-4 PMC8926922

[pbi13928-bib-0029] Goswami, R.S. and Kistler, H.C. (2004) Heading for disaster: *Fusarium graminearum* on cereal crops. Mol. Plant Pathol. 5, 515–525. 20565626 10.1111/j.1364-3703.2004.00252.x

[pbi13928-bib-0030] Gunupuru, L.R. , Perochon, A. and Doohan, F.M. (2017) Deoxynivalenol resistance as a component of FHB resistance. Trop. Plant Pathol. 42, 175–183.

[pbi13928-bib-0031] He, X. , Singh, P.K. , Dreisigacker, S. , Singh, S. , Lillemo, M. and Duveiller, E. (2016) Dwarfing genes Rht‐B1b and Rht‐D1b are associated with both Type I FHB susceptibility and low anther extrusion in two bread wheat populations. PLoS One, 11, e0162499. 27606928 10.1371/journal.pone.0162499 PMC5015901

[pbi13928-bib-0032] Holland, J. , Nyquist, W.E. and Cervantes‐Martinez, C.T. (2003) Estimating and interpreting heritability for plant breeding: An update. Plant Breed. Rev. 22, 9–111.

[pbi13928-bib-0033] Huang, S. , Sirikhachornkit, A. , Faris, J.D. , Su, X. , Gill, B.S. , Haselkorn, R. and Gornicki, P. (2002) Phylogenetic analysis of the acetyl‐CoA carboxylase and 3‐phosphoglycerate kinase loci in wheat and other grasses. Plant Mol. Biol. 48, 805–820. 11999851 10.1023/a:1014868320552

[pbi13928-bib-0034] Jansen, C. , von Wettstein, D. , Schäfer, W. , Kogel, K.‐H. , Felk, A. and Maier, F.J. (2005) Infection patterns in barley and wheat spikes inoculated with wild‐type and trichodiene synthase gene disrupted *Fusarium graminearum* . Proc. Natl Acad. Sci. USA 102, 16892–16897. 16263921 10.1073/pnas.0508467102 PMC1283850

[pbi13928-bib-0035] Kassambra A (2019) Ggpubr: ‘ggplot2’ Based Publication Ready Plots. R package version 0.2.4.

[pbi13928-bib-0036] Kishii, M. (2019) An update of recent use of aegilops species in wheat breeding. Front. Plant Sci. 10, 585. 31143197 10.3389/fpls.2019.00585 PMC6521781

[pbi13928-bib-0037] Klahr, A. , Zimmermann, G. , Wenzel, G. and Mohler, V. (2007) Effects of environment, disease progress, plant height and heading date on the detection of QTLs for resistance to Fusarium head blight in an European winter wheat cross. Euphytica, 154, 17–28.

[pbi13928-bib-0038] Kluger, B. , Bueschl, C. , Lemmens, M. , Michlmayr, H. , Malachova, A. , Koutnik, A. , Maloku, I. *et al*. (2015) Biotransformation of the mycotoxin deoxynivalenol in fusarium resistant and susceptible near isogenic wheat lines. PLoS One, 10, e0119656. 25775425 10.1371/journal.pone.0119656 PMC4361057

[pbi13928-bib-0039] Knutsen, H.K. , Alexander, J. , Barregård, L. , Bignami, M. , Brüschweiler, B. , Ceccatelli, S. , Cottrill, B. *et al*. (2017) Risks to human and animal health related to the presence of deoxynivalenol and its acetylated and modified forms in food and feed. EFSA J. 15, e04718. 32625635 10.2903/j.efsa.2017.4718 PMC7010102

[pbi13928-bib-0040] Lagudah, E.S. and Krattinger, S.G. (2019) A new player contributing to durable Fusarium resistance. Nat. Genet. 51, 1070–1071. 31253973 10.1038/s41588-019-0454-3

[pbi13928-bib-0041] Lemmens, M. , Scholz, U. , Berthiller, F. , Dall'Asta, C. , Koutnik, A. , Schuhmacher, R. , Adam, G. *et al*. (2005) The ability to detoxify the mycotoxin deoxynivalenol colocalizes with a major quantitative trait locus for Fusarium head blight resistance in wheat. Mol. Plant‐Microbe Interact. 18, 1318–1324. 16478051 10.1094/MPMI-18-1318

[pbi13928-bib-0042] Lemmens, M. , Steiner, B. , Sulyok, M. , Nicholson, P. , Mesterhazy, A. and Buerstmayr, H. (2016) Masked mycotoxins: does breeding for enhanced Fusarium head blight resistance result in more deoxynivalenol‐3‐glucoside in new wheat varieties? World Mycotoxin J. 9, 741–754.

[pbi13928-bib-0043] Li, T. , Bai, G. , Wu, S. and Gu, S. (2011) Quantitative trait loci for resistance to fusarium head blight in a Chinese wheat landrace Haiyanzhong. Theor. Appl. Genet. 122, 1497–1502. 21344182 10.1007/s00122-011-1549-0

[pbi13928-bib-0044] Li, X. , Shin, S. , Heinen, S. , Dill‐Macky, R. , Berthiller, F. , Nersesian, N. , Clemente, T. *et al*. (2015) Transgenic wheat expressing a barley UDP‐glucosyltransferase detoxifies deoxynivalenol and provides high levels of resistance to *Fusarium graminearum* . Mol. Plant‐Microbe Interact. 28, 1237–1246. 26214711 10.1094/MPMI-03-15-0062-R

[pbi13928-bib-0045] Li, X. , Michlmayr, H. , Schweiger, W. , Malachova, A. , Shin, S. , Huang, Y. , Dong, Y. *et al*. (2017) A barley UDP‐glucosyltransferase inactivates nivalenol and provides Fusarium Head Blight resistance in transgenic wheat. J. Exp. Bot. 68, 2187–2197. 28407119 10.1093/jxb/erx109 PMC5447872

[pbi13928-bib-0046] Li, G. , Jia, L. , Zhou, J. , Fan, J. , Yan, H. , Shi, J. , Wang, X. *et al*. (2019) Evaluation and precise mapping of QFhb.nau‐2B conferring resistance against Fusarium infection and spread within spikes in wheat (*Triticum aestivum* L.). Mol. Breed. 39, 62.

[pbi13928-bib-0047] Lin, F. , Xue, S.L. , Zhang, Z.Z. , Zhang, C.Q. , Kong, Z.X. , Yao, G.Q. , Tian, D.G. *et al*. (2006) Mapping QTL associated with resistance to Fusarium head blight in the Nanda2419 × Wangshuibai population. II: type I resistance. Theor. Appl. Genet. 112, 528–535. 16328229 10.1007/s00122-005-0156-3

[pbi13928-bib-0048] Liu, Y. , Salsman, E. , Fiedler, J.D. , Hegstad, J.B. , Green, A. , Mergoum, M. , Zhong, S. *et al*. (2019) Genetic mapping and prediction analysis of FHB resistance in a hard red spring wheat breeding population. Front. Plant Sci. 10, 1007. 31447872 10.3389/fpls.2019.01007 PMC6691880

[pbi13928-bib-0049] Löffler, M. , Schön, C.‐C. and Miedaner, T. (2009) Revealing the genetic architecture of FHB resistance in hexaploid wheat (*Triticum aestivum* L.) by QTL meta‐analysis. Mol. Breed. 23, 473–488.

[pbi13928-bib-0050] Luo, M.‐C. , Gu, Y.Q. , Puiu, D. , Wang, H. , Twardziok, S.O. , Deal, K.R. , Huo, N. *et al*. (2017) Genome sequence of the progenitor of the wheat D genome *Aegilops tauschii* . Nature, 551, 498–502. 29143815 10.1038/nature24486 PMC7416625

[pbi13928-bib-0051] Ma, H.‐X. , Bai, G.‐H. , Zhang, X. and Lu, W.‐Z. (2006) Main effects, epistasis, and environmental interactions of quantitative trait Loci for fusarium head blight resistance in a recombinant inbred population. Phytopathology, 96, 534–541. 18944314 10.1094/PHYTO-96-0534

[pbi13928-bib-0052] Maccaferri, M. , Harris, N.S. , Twardziok, S.O. , Pasam, R.K. , Gundlach, H. , Spannagl, M. , Ormanbekova, D. *et al*. (2019) Durum wheat genome highlights past domestication signatures and future improvement targets. Nat. Genet. 51, 885–895. 30962619 10.1038/s41588-019-0381-3

[pbi13928-bib-0053] McFadden, E.S. and Sears, E.R. (1946) The origin of Triticum spelta and its free‐threshing hexaploid relatives. J. Hered. 37, 107–116. 20985728 10.1093/oxfordjournals.jhered.a105590

[pbi13928-bib-0054] Mesterházy, Á. , Bartók, T. , Mirocha, C.G. and Komoróczy, R. (1999) Nature of wheat resistance to Fusarium head blight and the role of deoxynivalenol for breeding. Plant Breed. 118, 97–110.

[pbi13928-bib-0055] Michlmayr, H. , Varga, E. , Malachová, A. , Fruhmann, P. , Piątkowska, M. , Hametner, C. , Šofrová, J. *et al*. (2018) UDP‐glucosyltransferases from rice, brachypodium, and barley: substrate specificities and synthesis of type A and B trichothecene‐3‐O‐β‐d‐glucosides. Toxins, 10, 111. 29509722 10.3390/toxins10030111 PMC5869399

[pbi13928-bib-0056] Muhovski, Y. , Batoko, H. and Jacquemin, J.‐M. (2012) Identification, characterization and mapping of differentially expressed genes in a winter wheat cultivar (Centenaire) resistant to *Fusarium graminearum* infection. Mol. Biol. Rep. 39, 9583–9600. 22718510 10.1007/s11033-012-1823-5

[pbi13928-bib-0057] Mujeeb‐Kazi, A. (2001a) Intergeneric hybrids in wheat: Current status in CIMMYT. In International Triticeae IV Symposium( Hernandez, P. , Moreno, M.T. , Cubero, J. and Martin, A. , eds), pp. 261–264. Cordoba, Spain: Junta de Andalucia. Consejeria de Agricultura y Pesca.

[pbi13928-bib-0058] Mujeeb‐Kazi, A. (2001b) Synthetic hexaploids for bread wheat improvement. In International Triticeae IV Symposium( Hernandez, P. , Moreno, M.T. , Cubero, J. and Martin, A. , eds), pp. 193–199. Cordoba, Spain: Junta de Andalucia. Consejeria de Agricultura y Pesca.

[pbi13928-bib-0059] Oliver, R.E. , Cai, X. , Xu, S.S. , Chen, X. and Stack, R.W. (2005) Wheat‐Alien species derivatives: a novel source of resistance to fusarium head blight in wheat. Crop Sci. 45, 1353–1360.

[pbi13928-bib-0060] Pasquet, J.‐C. , Changenet, V. , Macadré, C. , Boex‐Fontvieille, E. , Soulhat, C. , Bouchabké‐Coussa, O. , Dalmais, M. *et al*. (2016) A Brachypodium UDP‐glycosyltransferase confers root tolerance to deoxynivalenol and resistance to Fusarium infection. Plant Physiol. 172, 559–574. 27378816 10.1104/pp.16.00371 PMC5074643

[pbi13928-bib-0061] Pestka, J. (2010) Toxicological mechanisms and potential health effects of deoxynivalenol and nivalenol. World Mycotoxin J. 3, 323–347.

[pbi13928-bib-0062] Pont, C. , Leroy, T. , Seidel, M. , Tondelli, A. , Duchemin, W. , Armisen, D. , Lang, D. *et al*. (2019) Tracing the ancestry of modern bread wheats. Nat. Genet. 51, 905–911. 31043760 10.1038/s41588-019-0393-z

[pbi13928-bib-0064] Poppenberger, B. , Berthiller, F. , Lucyshyn, D. , Sieberer, T. , Schuhmacher, R. , Krska, R. , Kuchler, K. *et al*. (2003) Detoxification of the Fusarium mycotoxin deoxynivalenol by a UDP‐glucosyltransferase from *Arabidopsis thaliana* . J. Biol. Chem. 278, 47905–47914. 12970342 10.1074/jbc.M307552200

[pbi13928-bib-0065] Rawat, N. , Pumphrey, M.O. , Liu, S. , Zhang, X. , Tiwari, V.K. , Ando, K. , Trick, H.N. *et al*. (2016) Wheat Fhb1 encodes a chimeric lectin with agglutinin domains and a pore‐forming toxin‐like domain conferring resistance to Fusarium head blight. Nat. Genet. 48, 1576–1580. 27776114 10.1038/ng.3706

[pbi13928-bib-0100] R Core Team . (2020) R: A language and environment for statistical computing. Vienna, Austria: R Foundation for Statistical Computing. Available from: https://www.R‐project.org/

[pbi13928-bib-0066] Sauerschnig, C. , Doppler, M. , Bueschl, C. and Schuhmacher, R. (2017) Methanol generates numerous artifacts during sample extraction and storage of extracts in metabolomics research. Metabolites, 8, 1. 29271872 10.3390/metabo8010001 PMC5875991

[pbi13928-bib-0068] Schroeder, H.W. and Christensen, J.J. (1963) Factors affecting resistance of Wheat to scab caused by *Gibberella zeae* . Phytopathology, 53, 831–838.

[pbi13928-bib-0069] Schweiger, W. , Boddu, J. , Shin, S. , Poppenberger, B. , Berthiller, F. , Lemmens, M. , Muehlbauer, G.J. *et al*. (2010) Validation of a candidate deoxynivalenol‐inactivating UDP‐glucosyltransferase from barley by heterologous expression in yeast. Mol. Plant‐Microbe Interact. 23, 977–986. 20521959 10.1094/MPMI-23-7-0977

[pbi13928-bib-0070] Schweiger, W. , Steiner, B. , Ametz, C. , Siegwart, G. , Wiesenberger, G. , Berthiller, F. , Lemmens, M. *et al*. (2013) Transcriptomic characterization of two major Fusarium resistance quantitative trait loci (QTLs), Fhb1 and Qfhs.ifa‐5A, identifies novel candidate genes. Mol. Plant Pathol. 14, 772–785. 23738863 10.1111/mpp.12048 PMC3902993

[pbi13928-bib-0071] Shimizu, K.K. , Copetti, D. , Okada, M. , Wicker, T. , Tameshige, T. , Hatakeyama, M. , Shimizu‐Inatsugi, R. *et al*. (2021) De novo genome assembly of the Japanese wheat cultivar norin 61 highlights functional variation in flowering time and Fusarium‐resistant genes in East Asian genotypes. Plant Cell Physiol. 62, 8–27. 33244607 10.1093/pcp/pcaa152 PMC7991897

[pbi13928-bib-0072] Srinivasachary, G.N. , Steed, A. , Hollins, T.W. , Bayles, R. , Jennings, P. and Nicholson, P. (2009) Semi‐dwarfing Rht‐B1 and Rht‐D1 loci of wheat differ significantly in their influence on resistance to Fusarium head blight. Theor. Appl. Genet. 118, 695–702. 19034409 10.1007/s00122-008-0930-0

[pbi13928-bib-0073] Su, Z. , Bernardo, A. , Tian, B. , Chen, H. , Wang, S. , Ma, H. , Cai, S. *et al*. (2019) A deletion mutation in TaHRC confers Fhb1 resistance to Fusarium head blight in wheat. Nat. Genet. 51, 1099–1105. 31182809 10.1038/s41588-019-0425-8

[pbi13928-bib-0074] Szabo‐Hever, A. , Zhang, Q. , Friesen, T.L. , Zhong, S. , Elias, E.M. , Cai, X. , Jin, Y. *et al*. (2018) Genetic diversity and resistance to fusarium head blight in synthetic hexaploid wheat derived from *Aegilops tauschii* and diverse *Triticum turgidum* Subspecies. Front. Plant Sci., 9. 30619402 10.3389/fpls.2018.01829 PMC6298526

[pbi13928-bib-0075] Venske, E. , Dos Santos, R.S. , Farias, D.R. , Rother, V. , da Maia, L.C. , Pegoraro, C. and Costa de Oliveira, A. (2019) Meta‐analysis of the QTLome of Fusarium head blight resistance in bread wheat: refining the current puzzle. Front. Plant Sci. 10, 727. 31263469 10.3389/fpls.2019.00727 PMC6585393

[pbi13928-bib-0076] Walkowiak, S. , Gao, L. , Monat, C. , Haberer, G. , Kassa, M.T. , Brinton, J. , Ramirez‐Gonzalez, R.H. *et al*. (2020) Multiple wheat genomes reveal global variation in modern breeding. Nature, 588, 277–283. 33239791 10.1038/s41586-020-2961-x PMC7759465

[pbi13928-bib-0077] Wang, Y.Z. and Miller, J.D. (1988) Effects of *Fusarium graminearum* metabolites on wheat tissue in relation to Fusarium head blight resistance. J. Phytopathol. 122, 118–125.

[pbi13928-bib-0078] Wang, H. , Sun, S. , Ge, W. , Zhao, L. , Hou, B. , Wang, K. , Lyu, Z. *et al*. (2020) Horizontal gene transfer of Fhb7 from fungus underlies Fusarium head blight resistance in wheat. Science, 368, eaba5435. 32273397 10.1126/science.aba5435

[pbi13928-bib-0079] Yan, W. , Li, H.B. , Cai, S.B. , Ma, H.X. , Rebetzke, G.J. and Liu, C.J. (2011) Effects of plant height on type I and type II resistance to fusarium head blight in wheat. Plant Pathol. 60, 506–512.

[pbi13928-bib-0080] Yang, J. , Bai, G. and Shaner, G.E. (2005) Novel quantitative trait loci (QTL) for Fusarium head blight resistance in wheat cultivar Chokwang. Theor. Appl. Genet. 111, 1571–1579. 16172894 10.1007/s00122-005-0087-z

[pbi13928-bib-0081] Yu, J.‐B. , Bai, G.‐H. , Zhou, W.‐C. , Dong, Y.‐H. and Kolb, F.L. (2008) Quantitative trait loci for Fusarium head blight resistance in a recombinant inbred population of Wangshuibai/Wheaton. Phytopathology, 98, 87–94. 18943242 10.1094/PHYTO-98-1-0087

[pbi13928-bib-0082] Zhou, Y. , Zhao, X. , Li, Y. , Xu, J. , Bi, A. , Kang, L. , Xu, D. *et al*. (2020) Triticum population sequencing provides insights into wheat adaptation. Nat. Genet. 52, 1412–1422. 33106631 10.1038/s41588-020-00722-w

[pbi13928-bib-0083] Zhou, Y. , Bai, S. , Li, H. , Sun, G. , Zhang, D. , Ma, F. , Zhao, X. *et al*. (2021) Introgressing the Aegilops tauschii genome into wheat as a basis for cereal improvement. Nat. Plants 7, 774–786. 34045708 10.1038/s41477-021-00934-w

