# Supplementary material for: Identification of a UDP‐glucosyltransferase conferring deoxynivalenol resistance in Aegilops tauschii and wheat
Source: Plant Biotechnol J. 2022 Oct 13;21(1):109–21. doi: 10.1111/pbi.13928 (PMC9829400; doi:10.1111/pbi.13928)
Supplement: Supplementary file 1 — Figure S1 Pearson's correlation coefficients of the Aegilops tauschii accessions for Fusarium head blight and deoxynivalenol severities and other traits. Figure S2 k‐mer‐based association genetics for Fusarium head blight severity. Figure S3 Amino acid alignment of the allelic diversity at the UGT for the sequenced Ae. tauschii accessions and wheat cv. Chinese Spring. Figure S4 Amino acid alignment of the chromosome ‘5D UGTs’ and the homoeologous proteins at chromosomes 5A and 5B of Chinese Spring. Figure S5 Sequence of the codon‐optimized Ae. tauschii UGT AET5Gv20385300, the deduced protein sequence and primers used for in vitro mutagenesis. Figure S6 Functional evaluation of the homoeologous uridine diphosphate (UDP)‐glucosyltransferases (UGT) at chromosomes 5A (TraesCS5A01G149600), 5B (TraesCS5B01G148300) and 5D (TraesCS5D01G143300, AET5Gv20385300). Figure S7 Sequences of the codon‐optimized UGTs TraesCS5A01G149600 and TraesCS5B01G148300. [file PBI-21-109-s002.docx]

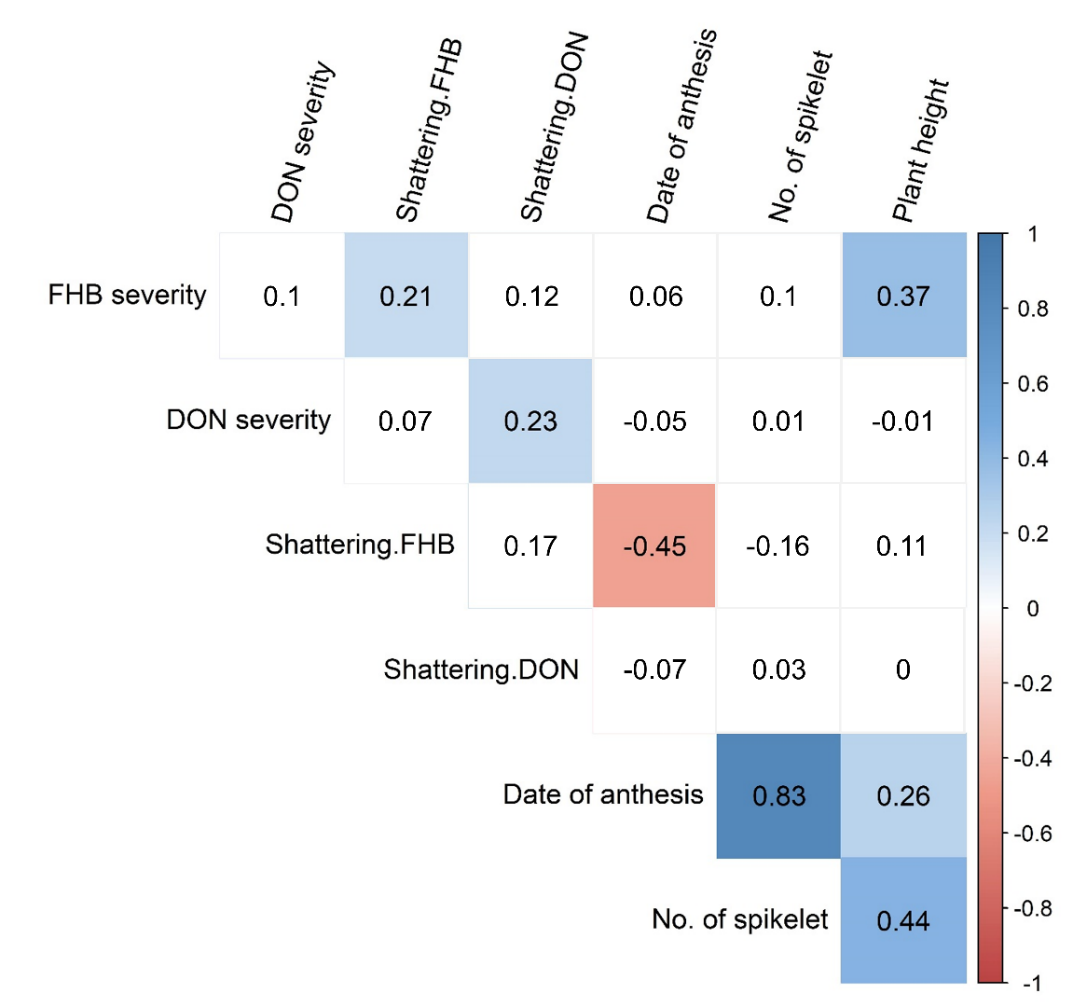


**Figure S1** Pearson’s correlation coefficients of the 147 *Aegilops tauschii* accessions for best linear unbiased estimates (BLUEs) across experiments among Fusarium head blight (FHB) and deoxynivalenol (DON) severities, incidence for seed shattering in the FHB and DON trials, date of anthesis (in days after December 1^st^), number of spikelets per spike and plant height. Significant correlations (*P* < 0.05) are highlighted in blue (positive correlation) or red (negative correlation).


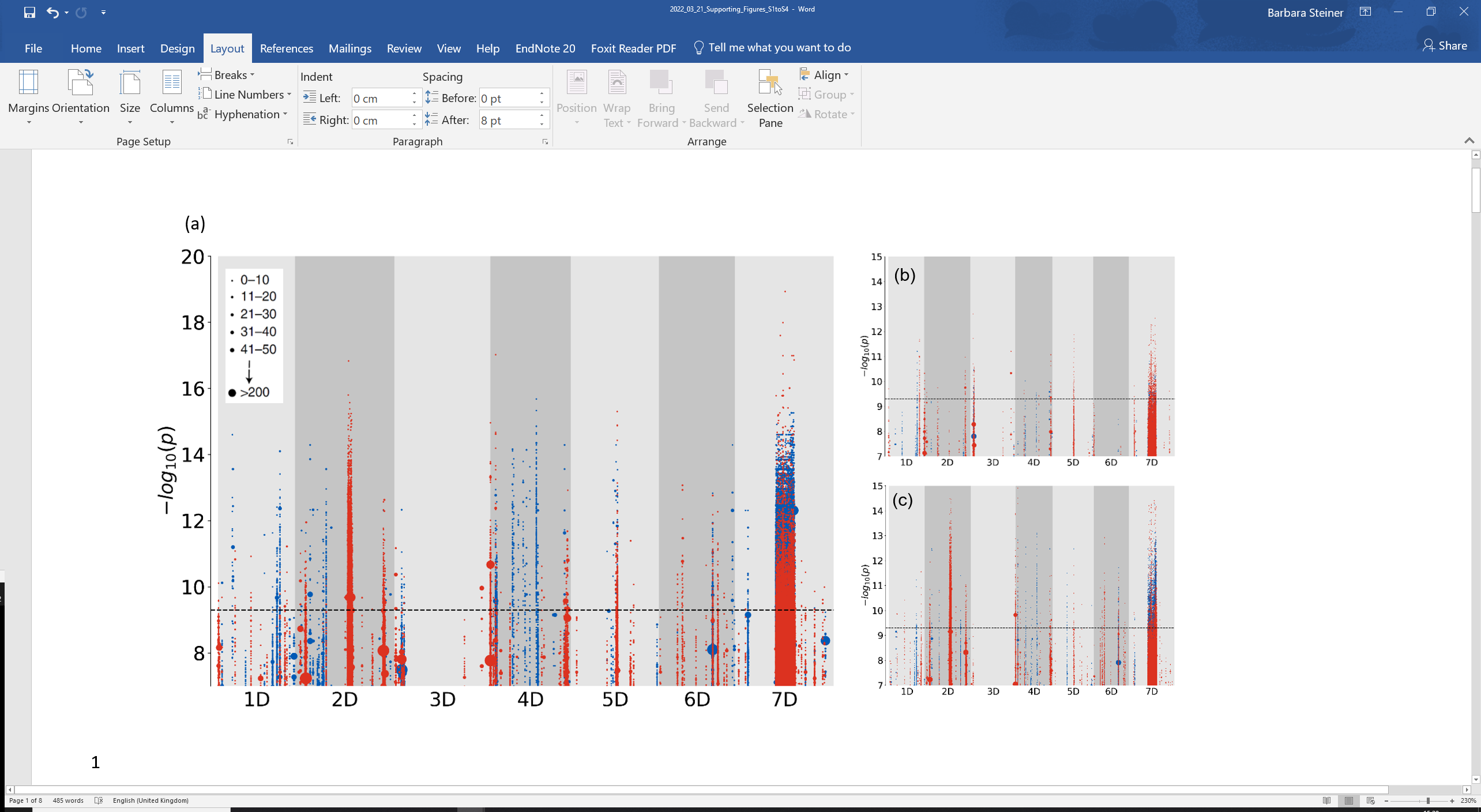


**Figure S2** *k*-mer-based association genetics for Fusarium head blight (FHB) severity of best linear unbiased estimates (BLUEs) (a) across experiments, (b) 2019 and (c) 2020 experiments mapped to the *Aegilops tauschii* reference genome AL8/78 partitioned to chromosomes 1D to 7D. Points on the plot illustrate *k*-mers associated with FHB resistance (blue) and FHB susceptibility (red). -log_10_ p-value = 9.3 was taken as the Bonferroni-adjusted threshold for evaluating the statistical significance of an association. Point size is proportional to the number of *k*-mers with a specific -log_10_ p-value (see insert).


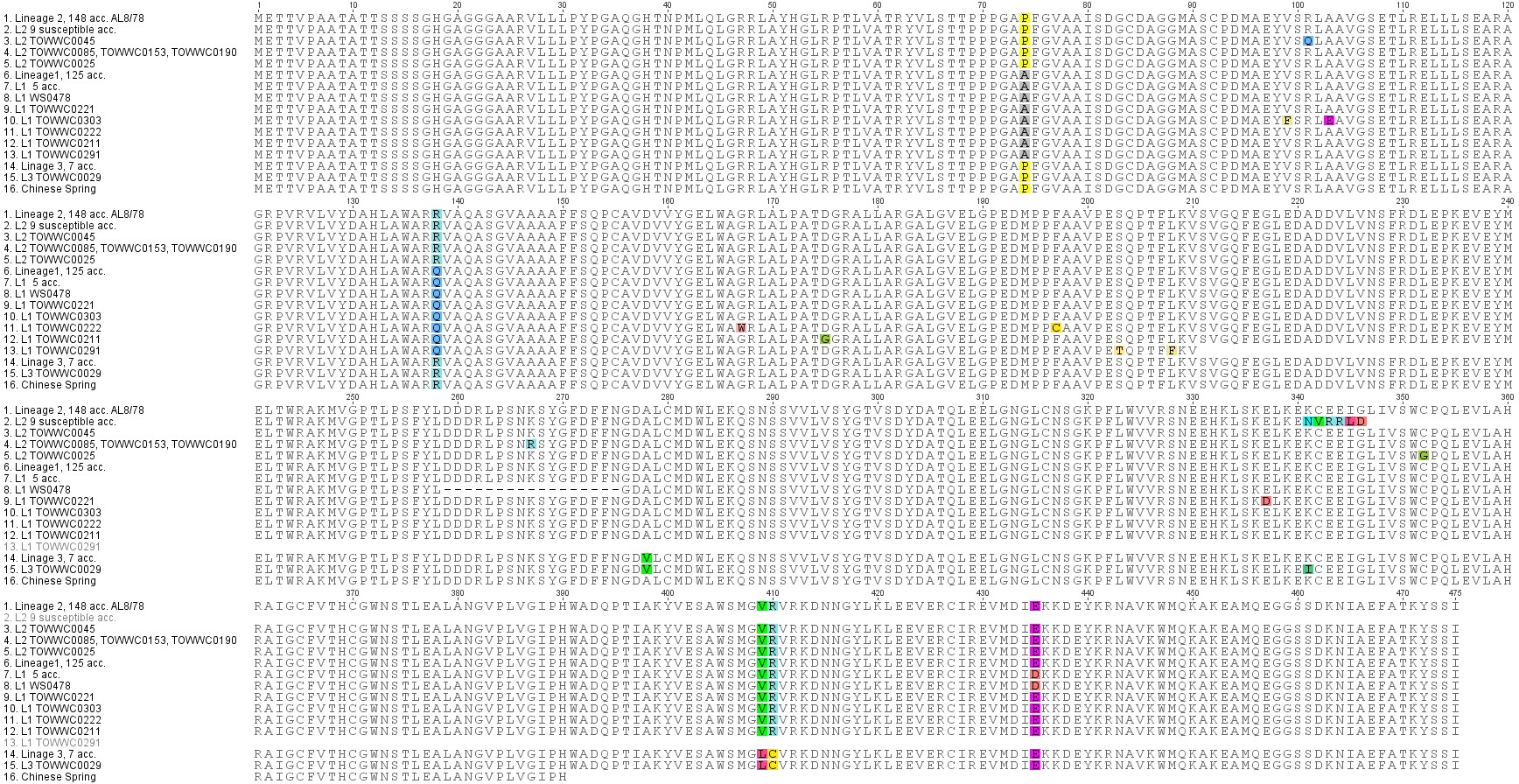


**Figure S3** Amino acid alignment of the allelic diversity at the uridine diphosphate (UDP)-glucosyltransferase (UGT) (*AET5Gv20385300)* for the sequenced *Aegilops tauschii* accessions and wheat cv. Chinese Spring. Sixteen UGT alleles were identified, whereas alleles (1.), (6.) and (14.) were the most common for lineages 2, 1 and 3 respectively. (1.) AL8/78 reference lineage 2 allele was identified for 148 accessions, (6.) 125 lineage 1 accessions harboured two nonsynonymous mutations (P74A; R138Q) in comparison to the reference sequence AL8/78 and (14.) seven lineage 3 accessions carried three amino acid changes (A278V; V409L; R410C). The complete list of the allelic status of all analysed *Ae. tauschii* accessions is available in Supporting Information Table S7.

**
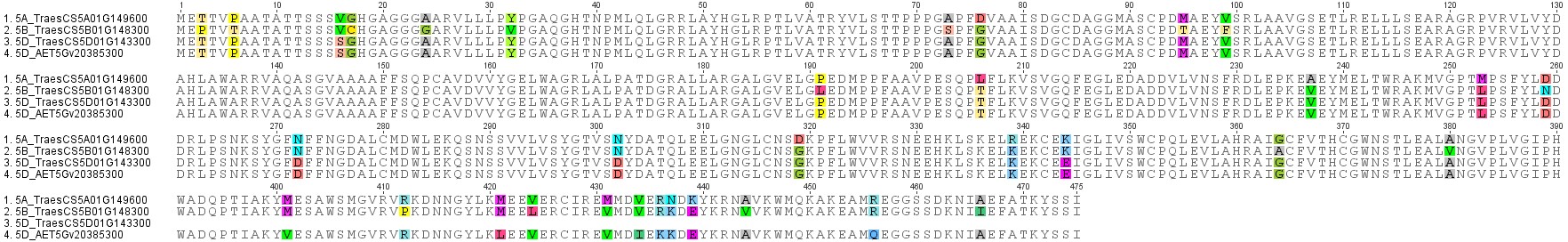
**

**Figure S4** Amino acid alignment of the chromosome 5D uridine diphosphate (UDP)-glucosyltransferase *TraesCS5D01G143300 and AET5Gv20385300* and the homoeologous proteins at chromosomes 5A (*TraesCS5A01G149600*) and 5B (*TraesCS5B01G148300*) of Chinese Spring.

(a) *Aegilops tauschii* UGT *AET5Gv20385300* codon-optimized sequence

AAGCTTATGGAAACCACCGTTCCGGCGGCTACCGCTACCACCTCTTCTTCTTCTGGTCATGGCGCAGGCGGCGGCGCAGCTCGTGTTCTGTTGCTGCCGTACCCGGGTGCGCAAGGTCATACCAATCCGATGCTGCAGCTGGGTCGTCGTCTGGCATACCATGGTCTGAGACCAACCCTGGTGGCGACCCGTTATGTTTTGAGCACCACCCCGCCGCCGGGTGCTCCATTTGGTGTTGCGGCTATTAGCGACGGTTGCGATGCGGGTGGTATGGCTAGCTGTCCGGATATGGCTGAATACGTGAGCCGTCTGGCAGCGGTTGGTAGCGAAACCCTGCGTGAACTGCTGCTGAGCGAAGCTCGTGCAGGTCGTCCGGTTCGTGTGCTGGTTTATGACGCTCATCTGGCGTGGGCTCGTCGTGTTGCACAAGCGAGCGGTGTTGCGGCGGCGGCGTTTTTCAGCCAGCCATGCGCTGTTGATGTTGTGTACGGTGAATTGTGGGCAGGTCGTCTGGCTTTGCCGGCTACCGATGGTAGAGCACTGCTGGCTCGTGGTGCTCTGGGTGTGGAACTGGGTCCGGAAGATATGCCGCCATTTGCAGCGGTTCCGGAAAGCCAACCGACCTTTCTGAAAGTGAGCGTTGGCCAGTTCGAAGGTCTGGAAGATGCAGATGACGTGCTGGTTAACAGCTTCCGTGACCTGGAACCGAAAGAAGTGGAATATATGGAACTGACCTGGCGTGCTAAGATGGTTGGTCCGACCCTGCCGAGCTTTTACCTGGATGACGATCGTCTGCCGAGCAACAAAtcaTATGGTTTCGACTTTTTCAACGGTGACGCGCTGTGCATGGACTGGCTGGAAAAGCAAAGCAATAGCAGCGTTGTGCTGGTGAGCTACGGTACCGTTAGCGACTATGATGCTACCCAGCTGGAAGAACTGGGTAATGGTCTGTGTAACAGCGGTAAACCGTTTCTGTGGGTTGTGCGTAGCAACGAAGAACATAAACTGAGCAAGGAACTGAAAGAAAAGTGCGAAGAAATTGGTCTGATCGTGAGCTGGTGTCCGCAACTGGAAGTTCTGGCACATCGTGCGATCGGTTGCTTCGTGACCCACTGTGGTTGGAATAGCACCCTGGAAGCTCTGGCAAACGGTGTGCCGCTGGTTGGTATTCCGCACTGGGCAGATCAGCCGACCATCGCGAAGTACGTTGAAAGCGCTTGGAGCATGGGTGTGCGTGTTCGTAAAGACAATAACGGTTATCTGAAGCTGGAAGAAGTGGAACGTTGTATCCGTGAAGTTATGGATATTGAAAAGAAAGATGAATACAAACGTAATGCGGTTAAGTGGATGCAAAAAGCTAAGGAAGCAATGCAAGAAGGCGGTAGCAGCGATAAGAACATTGCGGAGTTTGCCACCAAGTATTCCTCTATTtaaGCGGCCGC

(b) translated

KLMETTVPAATATTSSSSGHGAGGGAARVLLLPYPGAQGHTNPMLQLGRRLAYHGLRPTLVATRYVLSTTPPPGAPFGVAAISDGCDAGGMASCPDMAEYVSRLAAVGSETLRELLLSEARAGRPVRVLVYDAHLAWARRVAQASGVAAAAFFSQPCAVDVVYGELWAGRLALPATDGRALLARGALGVELGPEDMPPFAAVPESQPTFLKVSVGQFEGLEDADDVLVNSFRDLEPKEVEYMELTWRAKMVGPTLPSFYLDDDRLPSNKSYGFDFFNGDALCMDWLEKQSNSSVVLVSYGTVSDYDATQLEELGNGLCNSGKPFLWVVRSNEEHKLSKELKEKCEEIGLIVSWCPQLEVLAHRAIGCFVTHCGWNSTLEALANGVPLVGIPHWADQPTIAKYVESAWSMGVRVRKDNNGYLKLEEVERCIREVMDIEKKDEYKRNAVKWMQKAKEAMQEGGSSDKNIAEFATKYSSI

(c) PCR primer

Forward: ADHfw 5’-CATTGTTCTCGTTCCCTTTCTTCC-3’

Reverse: AetUGTdelAet_rv (*Ae. tauschii* susceptible):

5’- GGAACTGAAAGAAAATGTGAGAAGATTGGACTGA -3’

3’- CCTTGACTTTCTTTTACACTCTTCTAACCTGACT -5’

5’- ATTATTgcggccgcTCAGTCCAATCTTCTCACATTTTCTTTCAGTTCC -3’

AetUGTdelCS_rv (Chinese Spring):

5’- CGCTGGTTGGTATTCCGCACTAg -3’

3’- GCGACCAACCATAAGGCGTGATC -5’

5’- ATTATTGCGGCCGCTAGTGCGGAATACCAACCAGCG -3’

**Figure S5** Sequence of the codon-optimized *Aegilops tauschii* UGT *AET5Gv20385300* (a), the deduced protein sequence (b) and primers used for *in vitro* mutagenesis (c). Sequences highlighted in yellow are the recognition sites for the restriction enzymes (HindIII, NotI) used for cloning, while the sequences, which were used to create the truncated versions are marked in orange (susceptible *Ae. tauschii* accessions) or green (Chinese Spring), respectively. Primer binding sites are underlined. The forward primer (ADHfw) binds to the ADH1 promoter, while the reverse primers bind to the coding sequence, mutated nucleotides are printed in blue, the inserted stop codon (TAG) leading to the premature stop in Chinese Spring is double underlined.


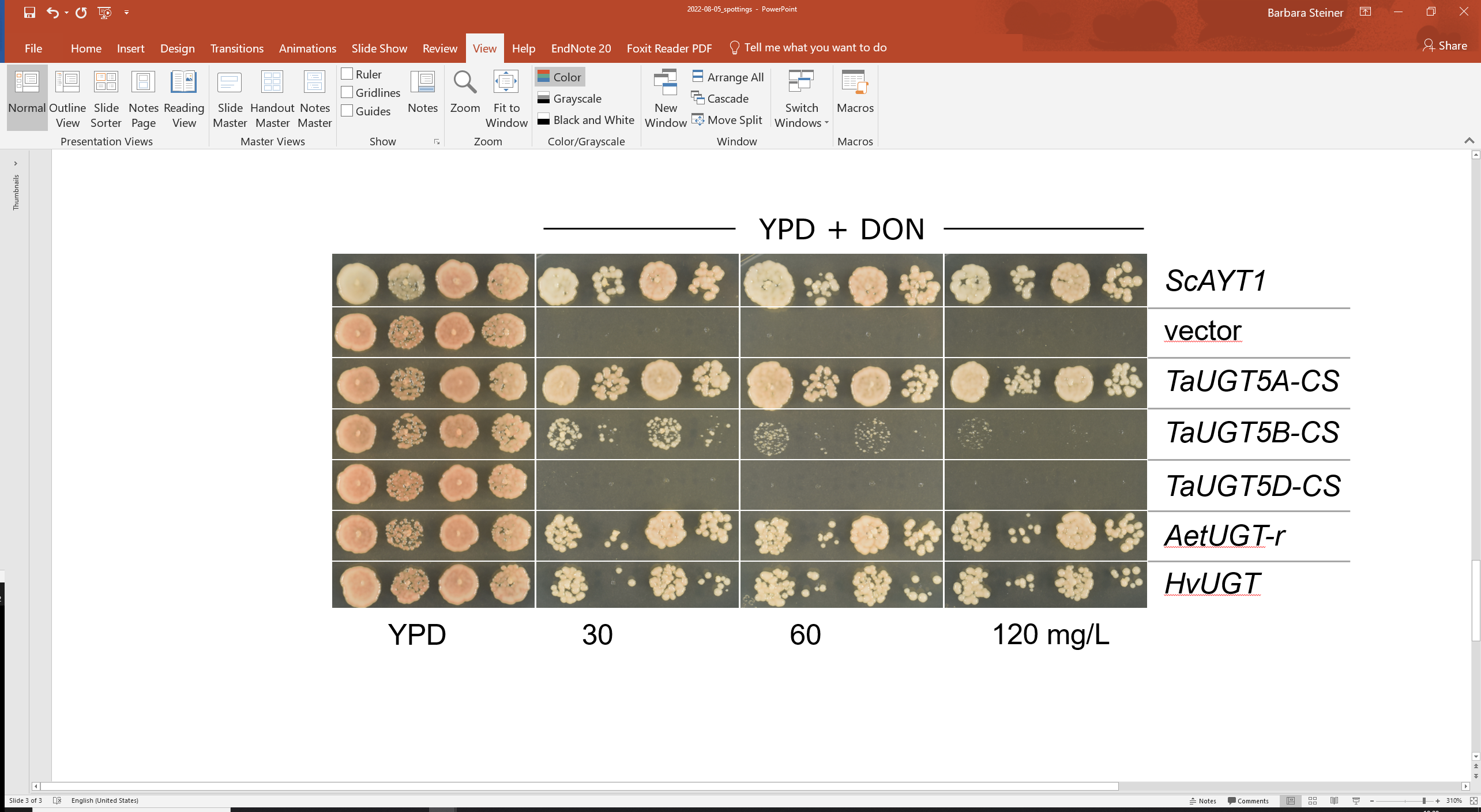
**Figure S6** Functional evaluation of the homoeologous uridine diphosphate (UDP)-glucosyltransferases (UGT) at chromosomes 5A (*TraesCS5A01G149600*), 5B (*TraesCS5B01G148300*) and 5D (*TraesCS5D01G143300*, *AET5Gv20385300*). A toxin-sensitive yeast strain was transformed with plasmids expressing the three homoeologous UGTs identified in Chinese Spring *TraesCS5A01G149600* (*TaUGT5A-CS*), *TraesCS5B01G148300* (*TaUGT5B-CS*), *TraesCS5D01G143300* (*TaUGT5D-CS*) and the UGT *AET5Gv20385300* (*AetUGT-r*) originally found in *Aegilops tauschii* but also present in wheat. The yeast acetyltransferase *ScAYT1* and the barley UGT *HvUGT13248* (*HvUGT*) serve as positive controls and the empty plasmid (vector) as negative control. Two individual transformants of each clone were grown in selective medium to mid-log phase, diluted to OD 0.3 and 0.03 and spotted onto yeast-extract-peptone-dextrose (YPD) media containing the indicated concentrations of deoxynivalenol (DON).

1. Chinese Spring UGT *TraesCS5A01G149600* codon-optimized sequence

ATGGAAACTACCGTCCCAGCTGCTACTGCTACCACTTCCTCATCCGTCGGTCACGGTGCCGGAGGTGGTGCCGCTAGAGTTTTGTTGCTACCATACCCAGGTGCTCAAGGTCATACCAACCCAATGTTGCAATTGGGTAGAAGATTGGCTTACCACGGTTTGAGACCAACTTTGGTTGCAACCCGTTACGTCCTATCCACAACTCCACCTCCAGGTGCTCCATTCGATGTCGCTGCCATCTCTGATGGTTGTGATGCTGGTGGTATGGCGTCTTGCCCAGACATGGCTGAATACGTTAGCAGACTAGCAGCCGTTGGTTCCGAAACCTTGAGAGAATTGTTGTTGTCTGAAGCTAGAGCCGGTAGACCAGTTCGTGTTTTGGTTTACGACGCTCATTTGGCTTGGGCCAGAAGAGTTGCTCAAGCCTCTGGTGTTGCTGCTGCTGCTTTCTTTTCTCAACCATGCGCTGTCGATGTCGTCTACGGTGAATTGTGGGCTGGTCGTCTTGCTTTGCCAGCTACTGACGGTCGTGCTTTATTAGCTAGAGGTGCTTTGGGTGTTGAATTGGGTCCAGAAGACATGCCACCTTTTGCCGCCGTTCCAGAATCCCAACCCTTGTTCTTGAAGGTTTCTGTCGGTCAATTCGAAGGTTTGGAAGATGCTGATGATGTTTTGGTTAACTCCTTCAGAGACTTGGAACCAAAGGAAGCTGAATACATGGAACTAACCTGGAGAGCTAAAATGGTTGGACCAACCATGCCATCTTTCTACTTGGACGATGACAGATTGCCATCTAACAAGTCTTACGGTTTCAACTTCTTCAACGGTGACGCTCTGTGTATGGACTGGTTGGAAAAGCAATCTAATTCCTCTGTTGTCTTAGTCAGTTATGGTACTGTATCCAACTACGATGCCACTCAATTAGAAGAATTGGGTAACGGTTTGTGTAACTCTGACAAGCCATTTTTATGGGTTGTCAGATCTAACGAAGAACACAAGTTGTCTAAGGAATTACGTGAAAAGTGTGAAAAGATCGGTTTAATTGTCTCTTGGTGTCCACAATTGGAAGTTCTGGCTCACAGAGCTATTGGTTGTTTCGTTACTCACTGTGGTTGGAACTCTACTTTGGAAGCCTTAGCCAATGGTGTCCCTTTGGTCGGCATCCCACACTGGGCTGACCAACCAACCATTGCCAAATATATGGAATCTGCTTGGTCCATGGGTGTCAGAGTCAGAAAGGACAACAACGGTTACTTGAAGATGGAAGAAGTTGAGAGATGTATCAGAGAAATGATGGACGTGGAAAGAAACGACAAATACAAGAGAAACGCTGTCAAGTGGATGCAAAAGGCCAAGGAAGCTATGAGAGAAGGTGGTTCTTCCGATAAGAACATTGCTGAATTCGCTACCAAGTACTCTTCCATCTGA

1. *TraesCS5A01G149600* translated

METTVPAATATTSSSVGHGAGGGAARVLLLPYPGAQGHTNPMLQLGRRLAYHGLRPTLVATRYVLSTTPPPGAPFDVAAISDGCDAGGMASCPDMAEYVSRLAAVGSETLRELLLSEARAGRPVRVLVYDAHLAWARRVAQASGVAAAAFFSQPCAVDVVYGELWAGRLALPATDGRALLARGALGVELGPEDMPPFAAVPESQPLFLKVSVGQFEGLEDADDVLVNSFRDLEPKEAEYMELTWRAKMVGPTMPSFYLDDDRLPSNKSYGFNFFNGDALCMDWLEKQSNSSVVLVSYGTVSNYDATQLEELGNGLCNSDKPFLWVVRSNEEHKLSKELREKCEKIGLIVSWCPQLEVLAHRAIGCFVTHCGWNSTLEALANGVPLVGIPHWADQPTIAKYMESAWSMGVRVRKDNNGYLKMEEVERCIREMMDVERNDKYKRNAVKWMQKAKEAMREGGSSDKNIAEFATKYSSI

1. Chinese Spring UGT *TraesCS5B01G148300* codon-optimized sequence

ATGGAACCAACTGTTACTGCTGCCACGGCCACTACTTCTTCTTCAGTCTGTCACGGTGCCGGTGGTGGCGGTGCTAGAGTCTTGTTGCTACCAGTCCCAGGTGCTCAAGGTCACACTAACCCAATGTTACAATTGGGTAGAAGATTAGCTTACCACGGTTTGAGACCAACCTTAGTCGCTACCAGATACGTTTTGTCCACCACCCCACCTCCTGGTTCTCCATTCGGTGTTGCCGCTATCTCTGATGGTTGTGATGCTGGTGGTATGGCCTCTTGTCCAGACACTGCTGAATACTTCAGCAGATTAGCTGCTGTCGGTTCCGAAACCTTGCGTGAATTGTTGTTGTCTGAAGCCAGAGCTGGTAGACCAGTTAGAGTTTTAGTTTACGACGCTCATTTGGCTTGGGCCCGTCGTGTTGCTCAAGCCTCTGGTGTTGCTGCCGCAGCTTTCTTTTCTCAACCATGCGCTGTTGACGTCGTCTACGGTGAATTGTGGGCTGGTCGTTTGGCTTTGCCAGCCACAGACGGAAGAGCGTTATTGGCTAGAGGTGCTTTGGGCGTAGAACTAGGTTTGGAAGACATGCCACCATTTGCTGCTGTTCCAGAATCCCAACCTACTTTCTTGAAGGTCTCTGTTGGTCAATTTGAAGGTCTGGAAGATGCTGATGATGTCTTGGTCAACTCTTTCAGAGACTTGGAACCAAAGGAAGTCGAGTATATGGAATTGACCTGGAGAGCCAAGATGGTTGGTCCAACTTTGCCATCCTTCTACTTGAACGATGACAGACTGCCATCTAACAAGTCTTACGGTTTCAACTTCTTCAATGGTGATGCTTTGTGTATGGACTGGTTGGAAAAGCAATCCAATTCTTCCGTTGTTTTGGTCTCCTATGGTACTGTGTCAAACTACGACGCCACTCAATTAGAAGAACTTGGTAACGGTTTATGTAACAGCGGTAAGCCATTCTTATGGGTTGTTAGATCCAACGAAGAACACAAGTTGTCCAAGGAATTAAAAGAAAAGTGTGAAAAAATCGGTTTGATTGTTTCTTGGTGCCCACAATTGGAAGTCTTGGCCCACAGAGCTATTGCTTGTTTCGTCACCCATTGTGGTTGGAACTCTACTTTGGAAGCCTTGGTTAACGGTGTTCCATTGGTCGGTATTCCACACTGGGCTGACCAACCAACCATTGCTAAGTACATGGAATCTGCTTGGTCTATGGGTGTCAGAGTTCCAAAGGACAACAACGGTTACTTGAAGATGGAAGAATTGGAAAGATGTATCAGAGAAGTTATGGATGTTGAAAGAAAGGATGAATACAAGAGAAACGTTGTCAAGTGGATGCAAAAGGCTAAAGAAGCTATGAGAGAAGGTGGTTCTTCTGACAAGAACATCATTGAATTCGCTACCAAGTACTCCTCCATCTGA

1. *TraesCS5B01G148300* translated

MEPTVTAATATTSSSVCHGAGGGGARVLLLPVPGAQGHTNPMLQLGRRLAYHGLRPTLVATRYVLSTTPPPGSPFGVAAISDGCDAGGMASCPDTAEYFSRLAAVGSETLRELLLSEARAGRPVRVLVYDAHLAWARRVAQASGVAAAAFFSQPCAVDVVYGELWAGRLALPATDGRALLARGALGVELGLEDMPPFAAVPESQPTFLKVSVGQFEGLEDADDVLVNSFRDLEPKEVEYMELTWRAKMVGPTLPSFYLNDDRLPSNKSYGFNFFNGDALCMDWLEKQSNSSVVLVSYGTVSNYDATQLEELGNGLCNSGKPFLWVVRSNEEHKLSKELKEKCEKIGLIVSWCPQLEVLAHRAIACFVTHCGWNSTLEALVNGVPLVGIPHWADQPTIAKYMESAWSMGVRVPKDNNGYLKMEELERCIREVMDVERKDEYKRNVVKWMQKAKEAMREGGSSDKNIIEFATKYSSI

**Figure S7** Sequences of the codon-optimized UGTs *TraesCS5A01G149600* (a, b) and *TraesCS5B01G148300* (c, d).
